# A new tuskless walrus from the Miocene of Orange County, California, with comments on the diversity and taxonomy of odobenids

**DOI:** 10.7717/peerj.5708

**Published:** 2018-10-12

**Authors:** Isaac Magallanes, James F. Parham, Gabriel-Philip Santos, Jorge Velez-Juarbe

**Affiliations:** 1 Department of Geological Sciences, California State University, Fullerton, Fullerton, CA, USA; 2 Department of Geological Sciences, University of Florida, Gainesville, FL, USA; 3 Raymond M. Alf Museum of Paleontology, Claremont, CA, USA; 4 Department of Mammalogy, Natural History Museum of Los Angeles County, Los Angeles, CA, USA; 5 Department of Paleobiology, National Museum of Natural History, Washington, DC, USA

**Keywords:** Odobenidae, Walrus, Phylogeny, Tusks, Pacific, Diversity, Neodobenia, Capistrano formation, Oso member, Miocene

## Abstract

We describe *Titanotaria orangensis* (gen. et. sp. nov.), a new species of walrus (odobenid) from the upper Miocene Oso Member of the Capistrano Formation of Orange County, California. This species is important because: (1) It is one of the best-known and latest-surviving tuskless walruses; (2) It raises the number of reported odobenid taxa from the Oso Member to four species making it one of the richest walrus assemblages known (along with the basal Purisima of Northern California); (3) It is just the second record of a tuskless walrus from the same unit as a tusked taxon. Our phylogenetic analysis places *T. orangensis* as sister to a clade that includes *Imagotaria downsi*, *Pontolis magnus*, *Dusignathus* spp., *Gomphotaria pugnax*, and Odobeninae. We propose new branch-based phylogenetic definitions for Odobenidae, Odobeninae, and a new node-based name (Neodobenia) for the clade that includes *Dusignathus* spp., *G. pugnax*, and Odobeninae. A richness analysis at the 0.1 Ma level that incorporates stratigraphic uncertainty and ghost lineages demonstrates maximum peaks of richness (up to eight or nine coeval lineages) near the base of Odobenidae, Neodobenia, and Odobenini. A more conservative minimum curve demonstrates that standing richness may have been much lower than the maximum lineage richness estimates that are biased by stratigraphic uncertainty. Overall the odobenid fossil record is uneven, with large time slices of the record missing on either side of the Pacific Ocean at some times and biases from the preserved depositional environments at other times. We recognize a provisional timescale for the transition of East Pacific odobenid assemblages that include “basal odobenids” (stem neodobenians) from the Empire and older formations (>7 Ma), to a mixture of basal odobenids and neodobenians from the Capistrano and basal Purisima (7–5 Ma), and then just neodobenians from all younger units (<5 Ma). The large amount of undescribed material will add new taxa and range extensions for existing taxa, which will likely change some of the patterns we describe.

## Introduction

**Figure 1:**
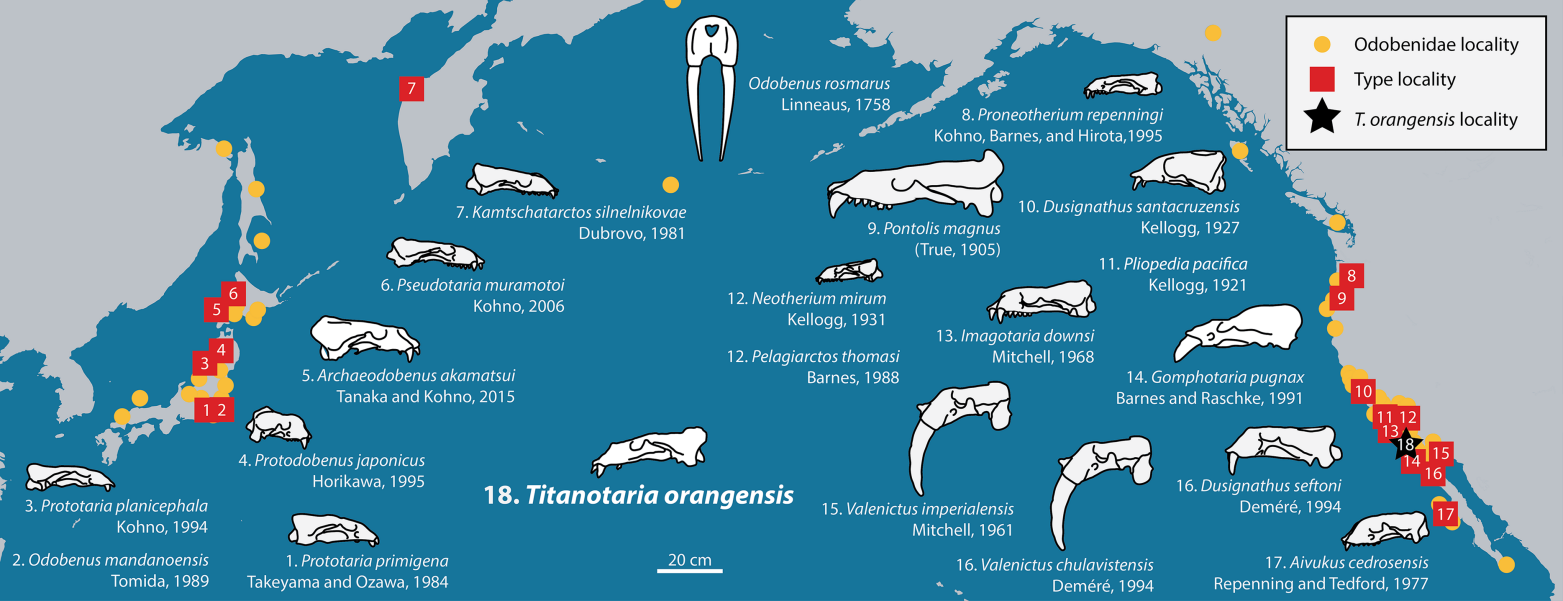
Distribution of fossil odobenid localities in the Pacific. Circles represent published localities. Numbered squares represent type localities. A star represents the locality of *Titanotaria orangensis* (OCPC 11141). Figured skulls are known from complete or partial cranial material and are drawn to scale referencing line drawings by Robert W. Boessenecker. The specimens facing right are found in the Western Pacific, while specimens facing left are found in the Eastern Pacific. Occurrence data and basemap were obtained from the Paleobiology Database using the following parameters: family = Odobenidae. Major contributors to this data set were John Alroy and Mark Uhen.

The modern walrus, *Odobenus rosmarus* (Linnaeus, 1758), is an iconic Arctic species and the last surviving member of a lineage that first appeared in the middle Miocene (∼16 Ma). For much of their evolutionary history, odobenids lacked the characteristic tusks, molluscivory, and Arctic distribution of *O. rosmarus*, but instead were much more diverse, widespread, and ecologically varied (Fig. 1). Most of the odobenid fossil record (until the Pliocene) is from the North Pacific, with 21 accepted species (Appendix 1) assigned to 18 genera. The high number of monotypic genera reflects a highly imbalanced, pectinate phylogeny. The majority of odobenid taxa lack diagnostic apomorphies, but rather fall along a continuum between generalized, ancestral pinnipedimorph taxa with many plesiomorphic characters (e.g., complex-crowned double-rooted teeth, no tusks, relatively small body size) and more *O. rosmarus*-like taxa (e.g., peg-like single-rooted teeth, tusks, relatively large body size; Boessenecker & Churchill, 2013; Tanaka & Kohno, 2015). But the history of odobenids is not as simple as that of an anagenetic lineage culminating in the modern species. For example, the most morphologically derived taxon is extinct (*Valenictus* Mitchell, 1961, lacks all teeth except its tusks). Also, multiple species of odobenid can be found in the same formation (Barnes, 1988; Deméré, 1994a; Tanaka & Kohno, 2015; Velez-Juarbe, in press) including even the coexistence of tusked and tuskless forms (Boessenecker, Perry & Schmitt, 2014: table 1).

**Figure 2:**
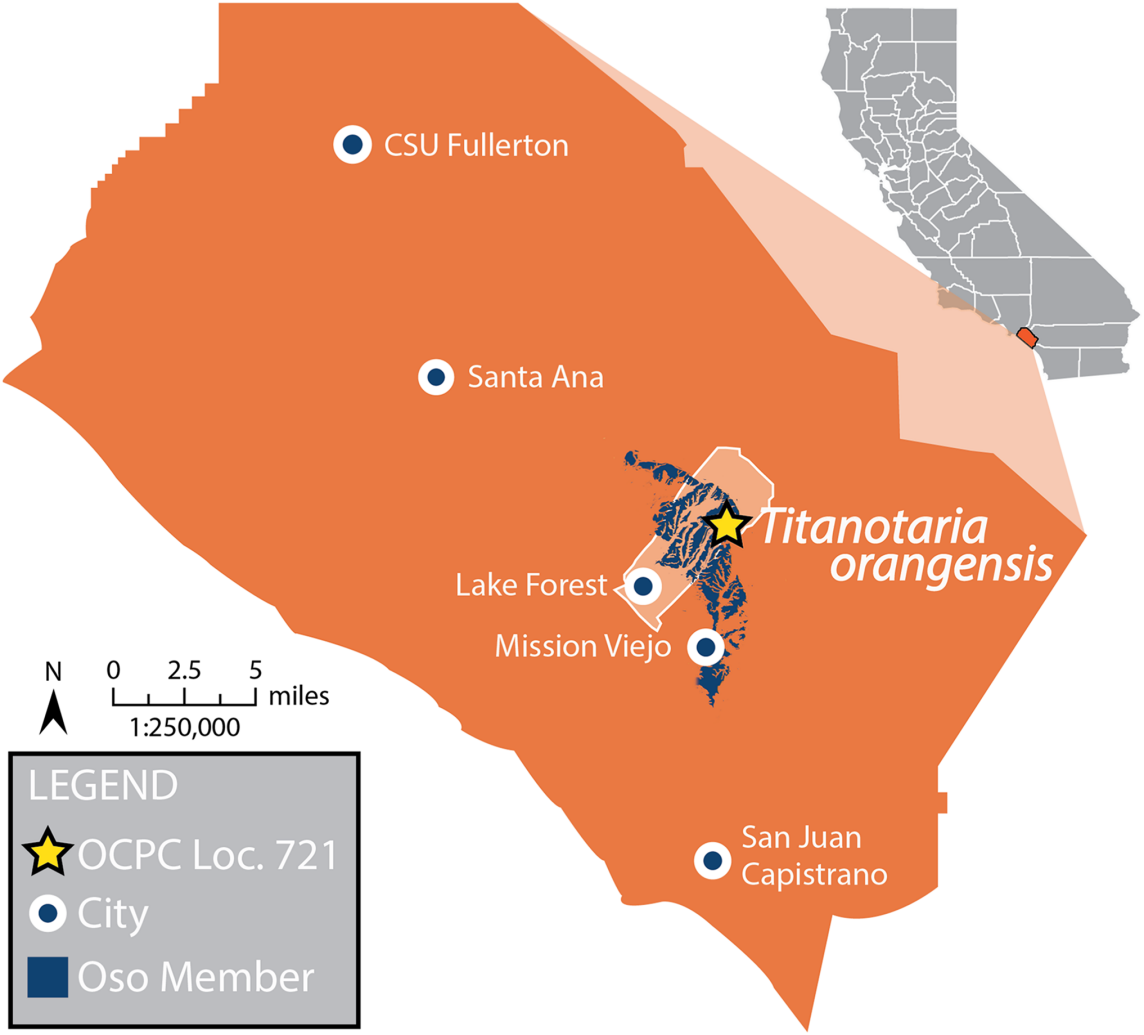
Map of Orange County, CA, and type locality for *Titanotaria orangensis*. Map of Orange County, California, showing where OCPC 11141 was discovered in the Oso Member (extent shown in blue) of the Capistrano Formation, Lake Forest (outline in white), Orange County (in orange), California. A star represents the locality (OCPC Locality 721) of *Titanotaria orangensis* (OCPC 11141). Map modified from Barboza et al. (2017).

The evolution of odobenid assemblages has received some recent attention (Boessenecker, 2017; Velez-Juarbe, 2017; Boessenecker & Churchill, 2018), and whereas some patterns are beginning to emerge, those studies note the existence of many undescribed specimens that could change our understanding once studied. In this study, we describe a new specimen of tuskless odobenid (OCPC 11141) from the upper Miocene Oso Member of the Capistrano Formation from Lake Forest, Orange County, California (Fig. 2). Although it was discovered in 1992 and is one of the more complete odobenid specimens known, OCPC 11141 has never been figured or described. The only mention of this specimen in the peer-reviewed literature is as part of a faunal list for the Oso Member (Barboza et al., 2017). This specimen is significant because it represents one of the best-known and latest-surviving tuskless odobenids, and therefore provides important data for the phylogeny of odobenids as well as the evolution of pinniped assemblages in the East Pacific.

In addition to describing OCPC 11141 and naming a new genus and species, we perform a phylogenetic analysis that we use as the basis for assessing odobenid phylogenetic taxonomy and lineage richness through time. For the richness analysis, we incorporate stratigraphic uncertainty and phylogenetic relationships (ghost lineages) across 0.1 Ma intervals (*n* = 173). We use the results of these analyses to present an overview of the odobenid fossil record, review the timing and recently proposed causes for richness changes, and highlight biases that should affect interpretations.

## Materials and Methods

### Material examined

*Aivukus cedrosensis* Repenning & Tedford, 1977 (LACM 154671, cast of type); *Dusignathus santacruzensis* Kellogg, 1927 (LACM 1527, cast of type; LACM 3011, 4342); *Dusignathus seftoni* Deméré, 1994a (LACM 155310, cast of type; LACM 135545, cast of SDNHM 20801); *Gomphotaria pugnax* Barnes & Raschke, 1991 (LACM 105151, 121508; LC 7750); *Imagotaria downsi* Mitchell, 1968 (LACM 144453, cast of type; USNM 23858, 184060); *Neotherium mirum* Kellogg, 1931 (LACM 4319, 52172, 98147, 123000, 123002, 127697; UCMP 81665); Odobenidae Allen, 1880 gen. et sp. indet. (LACM 135920); *Odobenus rosmarus* (LACM 31336, 52376, 52423, 72561); *Odobenus mandanoensis* (NMNS 18911); *Ontocetus* Leidy 1859 sp. (LACM 150001, cast of SFMCV-0001); *Pelagiarctos* Barnes, 1988 sp. (SDNHM 131041); *Pelagiarctos thomasi* Barnes, 1988 (LACM 121501); *Pliopedia pacifica* Kellogg, 1921 (LACM 57055, cast of type); *Pontolis magnus* True, 1905 (LACM 101151, cast of type); *Proneotherium* Barnes in Kohno, Barnes & Hirota 1995 sp. (LACM 128412); *Protodobenus japonicus* Horikawa, 1995 (LACM 140726, cast of type); *Prototaria planicephala* Kohno, 1994 (LACM 134826, cast of type); *Prototaria primigena* Takeyama & Ozawa, 1984 (LACM 130432, cast of type); *Valenictus imperialensis* Mitchell, 1961 (LACM 3926).

### Phylogenetic methods

We added *Titanotaria orangensis* to the 23 taxon, 90 character, data matrix of Boessenecker & Churchill (2013) with the addition of *Archaeodobenus akamatsui* Tanaka & Kohno, 2015 and character 91 from Tanaka & Kohno (2015). We analyzed the data set with all unordered characters as well as with some characters ordered. For the analysis with ordered characters, we identified 21 characters that have more than two-character states, form a plausible transformation series, and are phylogenetically informative for the Odobenidae. The ordered characters are 5, 9, 11, 33, 37, 51–53, 55, 56, 61, 62, 70, 71, 74, 76, 77, 80, 82, 83, and 86. For three characters (52, 71, 86) we changed the number applied to character states so that they could be easily analyzed as ordered (switched 0s and 1s). For character 5 we combine states 1 and 2 into state 1 “1 = foramina coalesced, 2 = trilobed pits” (in this case state 3 became state 2). The coding for character 4 matches that of the (Boessenecker & Churchill, 2013) matrix, which is different from that described by their character descriptions (in which the descriptions for states for 2 and 1 are reversed). Also, following their character guide text we change the coding of *I. downsi* from 0 to ? for this character 4. Character 17 included a state 3 for *Erignathus* and *Monachus*, but there is no state 3 described; we change this character to state 1 for these taxa. For *I. downsi*, we updated character 48 (from 0 to 1) based on Repenning & Tedford (1977; USNM 23858). For *N. mirum* we updated character 59 (from 0 to 1) based on Velez-Juarbe (in press). Character 61 was updated for *G. pugnax* (from 1 to 0) based on the type. Character 79 was coded as state 0 for *Callorhinus ursinus* (Linnaeus, 1758) and *Allodesmus kernensis* Kellogg, 1922 by Boessenecker & Churchill (2013), but is corrected here to 1. We include the character codings for *P. magnus* that were included in Boessenecker & Churchill (2013) and Tanaka & Kohno (2015) while noting that these character states have not yet been described.

We analyzed the data matrix (Data S1) in PAUP* 4.0a build 159 (Swofford, 2002) using a heuristic search algorithm with 1,000 stepwise additions. We assessed support for each node with a bootstrap analysis of 1,000 replicates with 100 addition sequence replicates and Bremer decay indices.

### Taxonomy

The electronic version of this article in portable document format will represent a published work according to the International Commission on Zoological Nomenclature (ICZN), and hence the new names contained in the electronic version are effectively published under that Code from the electronic edition alone. This published work and the nomenclatural acts it contains have been registered in ZooBank, the online registration system for the ICZN. The ZooBank LSIDs (Life Science Identifiers) can be resolved and the associated information viewed through any standard web browser by appending the LSID to the prefix http://zoobank.org/. The LSID for this publication is: LSIDurn:lsid:zoobank.org:pub:7826C97C-3994-4519-89F0-603E6135F3DB The online version of this work is archived and available from the following digital repositories: PeerJ, PubMed Central, and CLOCKSS.

### Richness curve methods

A time constrained richness curve was constructed to show the minimum, maximum count of known and inferred odobenid lineages (richness) present throughout their history, taking into account chronostratigraphic uncertainty and ghost lineages. For each taxon, we estimated a minimum and maximum possible age of occurrence by performing a literature review of chronostratigraphic data to build on estimates presented by recent studies (Boessenecker & Churchill, 2015; Barboza et al., 2017; Kloess & Parham, 2017; Velez-Juarbe, 2017; Boessenecker & Churchill, 2018). We exclude the recently described *Nanodobenus arandai* Velez-Juarbe & Salinas-Márquez, 2018, because it appeared late in the review process for this paper and is the least chronostratigraphically constrained odobenid taxon (15.7 and 9.2 Ma, 6.5 Ma range is more than double that of any other taxon known from just one site). Detailed explanations for the age ranges of all taxa are included in Appendix 1. We used these data and the phylogenetic trees from our preferred hypothesis to generate a stratigraphically-constrained cladogram which was used to count the minimum and maximum number of lineages at 0.1 Ma time slices (0.0–17.3 Ma, 173 time slices).

The maximum lineage count was determined by assuming that each taxon spanned from the oldest to the youngest possible age of the rock units that include referred specimens. Six taxa are known from multiple formations (*I. downsi*, *D. santacruzensis*, *Ontocetus emmonsi* Leidy, 1859, *Pelagiarctos* [incl. *P. thomasi*], *P. pacifica*, *V. imperialensis*) and so the maximum age range of both units was used for their broadest possible age range. The broadest possible age range of four presumably valid taxa that are not included in the cladistic analysis (*Odobenus mandanoensis* Tomida, 1989, *P. pacifica*, *P. planicephala*, *V. imperialensis*) were included in the maximum count. We also extended the maximum age range of the *Valenictus* lineage based on Boessenecker (2017).

The minimum lineage count was generated by assuming originations and extinctions within the possible range that gave the lowest possible number of lineages at any given time. For the most part this involved counting ghost lineages since, at any time within the actual estimate range, a taxon can be presumed to be extinct or unoriginated. However, two taxa are known from more than one formation that do not overlap in time (*I. downsi*, *O. emmonsi*) providing a “known age range” for those taxa that was always included in the minimum count.

Maximum and minimum lineage counts from the nine trees from the ordered character analysis that varied among the ingroup were compared, and the maximum and minimum age estimates for each 0.1 Ma bin among the trees was included in the curves. We did not include undescribed or insufficiently characterized specimens that have been mentioned in recent papers (Appendix 2). By excluding these specimens we bias our richness estimates downward and so comparisons with other studies must be evaluated in this context. Nevertheless, this exercise is useful as an exploration of other factors that influence estimates of odobenid richness, as well as an estimate from the more established part of the record that can be used as a baseline for future studies.

In addition to the maximum and minimum global curves, we also generated a curve based on maximum lineage counts from an analysis where all taxa from outside the east Pacific were pruned from the trees. The purpose of this curve is to demonstrate the relative contribution of the East Pacific sites to the fossil record of odobenids.

## Systematic Paleontology

MAMMALIA Linnaeus, 1758
CARNIVORA Bowdich, 1821
ODOBENIDAE Allen, 1880
*TITANOTARIA* gen. nov.

**Type and included species**—*Titanotaria orangensis* sp. nov.

**Etymology**—*Titan*- for the nickname of California State University, Fullerton, the Titans, to recognize the partnership with the County of Orange (Orange County Parks) that formed the John D. Cooper Archaeology and Paleontology Center; -*otaria* for *Otaria* Péron, 1816 the generotypus for the Otariidae, from which other fossil odobenid names have been derived, for example, *Imagotaria* Mitchell, 1968, *Gomphotaria* Barnes & Raschke, 1991, *Prototaria* Takeyama & Ozawa, 1984, and *Pseudotaria* Kohno, 2006.

**Diagnosis**—as for species.

*TITANOTARIA ORANGENSIS* sp. nov.
Figs. 3–13; Tables 1–7
“Odobenidae sp. A” Barboza et al. (2017: 2, 15)

**Holotype**—OCPC 11141, a male individual, including a skull, mandible, and postcrania including a nearly complete appendicular and axial skeleton. The missing elements include parts of the right scapula, humerus, and radius, as well as some ribs, some of the autopodia, and most of the pelvis.

**Etymology**—*orange*- for the County of Orange, California, USA, where the holotype was found; -*ensis* for the Latin word meaning “originating in’ (i.e., from).

**Locality**—The type specimen was found at OCPC Locality 721, in Lake Forest, Orange County, California, during paleontological mitigation for the construction of the Foothill Transportation Corridor (California State Route 241). Collected by David Alexander, July 9, 1993.

**Horizon**—The type specimen comes from the Oso Member of the upper Miocene Capistrano Formation. The Oso Member is a nearshore facies with fossils of marine and terrestrial vertebrates. A preliminary list of the Oso Member assemblage can be found in Barboza et al. (2017) and includes at least three other putative taxa of walruses, as well as sea cows, whales, desmostylians, sea turtles, crocodylians, sharks, fish, birds, elephants, rhinos, and horses. Using biostratigraphic correlations, Barboza et al. (2017) constrained the age of the Oso Member to 6.6–5.8 Ma.

**Referred specimen and locality**—LACM 160199, anterior end of right mandible, missing all teeth except the base of the canine. LACM Loc. 4438, Lake Mission Viejo, Mission Viejo, Orange Co., CA; collected by L.G. Barnes, August 4, 1978.

**Diagnosis**—*Titanotaria orangensis* can be diagnosed from neodobenians (see Phylogenetic Taxonomy) by having a short mandibular symphysis, C1/c1 that are proportional in size, lingual cingulum well-developed on P1 and P2, double-rooted p2–4 and double-rooted m1. *Titanotaria orangensis* can be distinguished from neodobenians and *P. magnus* by having postcanine crowns with enamel, a posterior crista on c1, paraconid cusps on the lower post-canines, and a double-rooted P4. *Titanotaria orangensis* can be distinguished from *I. downsi*, *P. magnus*, and neodobenians by having a double-rooted P3. It can be distinguished from *I. downsi* and *Pelagiarctos* sp. by having a mandibular symphysis that is less than 50% the length of the horizontal ramus. *Titanotaria orangensis* can be further distinguished from *I. downsi* by having a ventral tuberosity of the zygomatic root, from *Pelagiarctos* sp. by having a sinuous ventral margin of the mandible, and from *P. thomasi* by having an unfused mandibular symphysis. *Titanotaria orangensis* can be distinguished from all other known odobenids (excluding *I. downsi*, *P. magnus*, neodobenians) by having a non-converging posterior nasal suture, short glenoid fossa, and a large mastoid process.

## Description

The type specimen (OCPC 11141) consists of a cranium, mandibles, and associated postcrania including a nearly complete appendicular and axial skeleton. The missing elements include parts of the right scapula, humerus, and radius, as well as some ribs, some of the autopodia, and most of the pelvis. Well-preserved tightly fused cranial sutures and an associated baculum indicate that the holotype of *Titanotaria orangensis* is an adult male (suture age 35; Sivertsen, 1954), with an estimated body length of 3.3 m (based on cranial, mandibular and dental measurements; Churchill, Clementz & Kohno, 2015). This body length estimate for *T. orangensis* is similar to that of some undescribed odobenids from the Monterey Formation, *G. pugnax* from the Capistrano Formation, and *O. rosmarus* (Velez-Juarbe, 2017; Lydersen, 2018).

For this report, we describe the cranium, mandibles, and phylogenetically relevant postcranial elements (humerus, radius, scapholunar, astragalus, calcaneum, entocuneiform, metacarpal 1, and baculum) of OCPC 11141. We list the phylogenetic characters (see Phylogenetic methods) in our description when that area is being described following a modified convention of Domning (1994) and Parham & Pyenson (2010). We use the format (c. *n*^1^[*n*^2^]) where *n*^1^ corresponds to the character and *n*^2^ corresponds to the character state.

### Cranium

**Figure 3:**
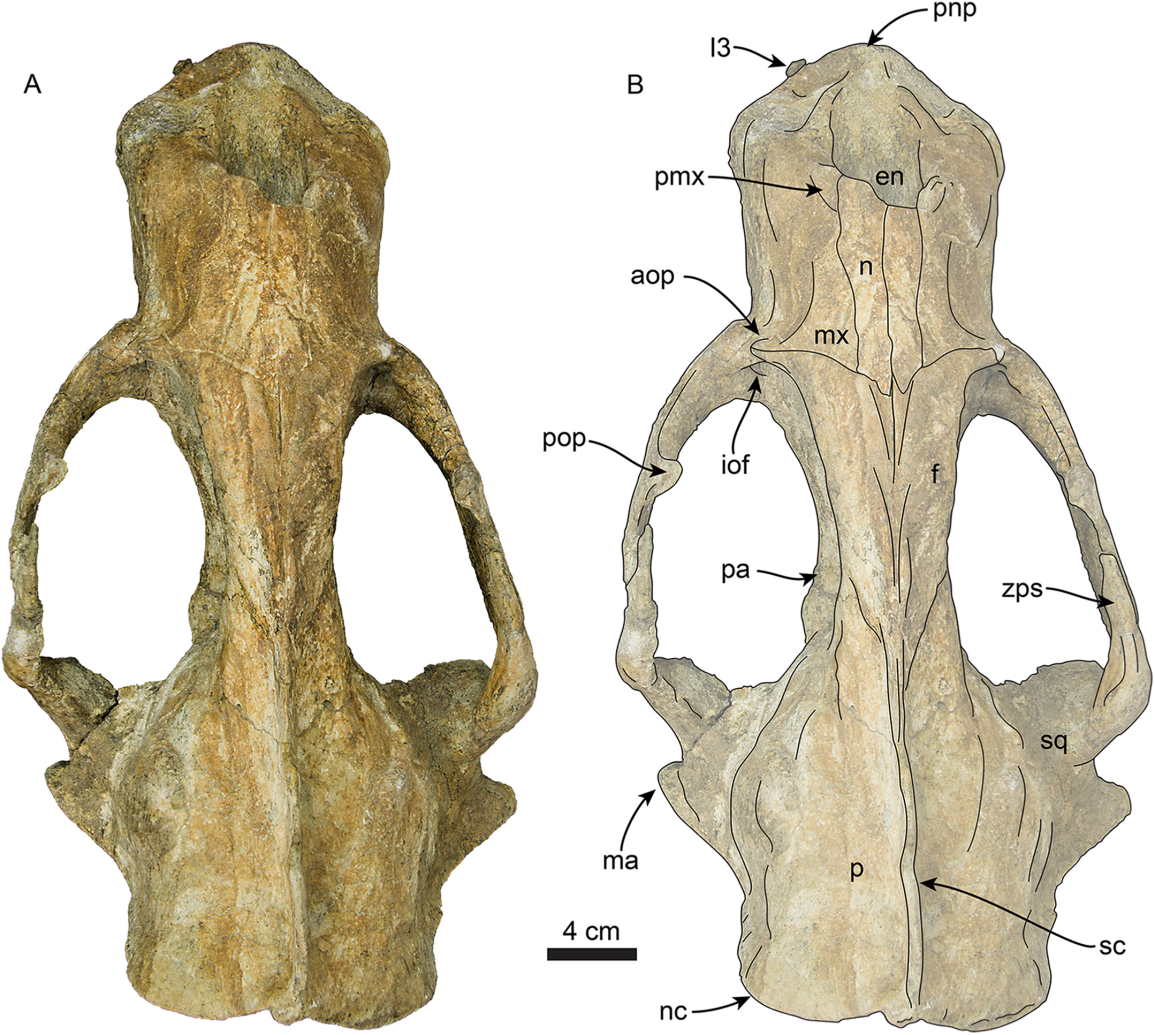
Skull of *Titanotaria orangensis* n. gen. et sp. (OCPC 11141). Skull in dorsal view, without labels (A) and labelled (B). Abbreviations: aop, antorbital process; en, external nares; f, frontal; I3, upper third incisor; iof, infraorbital foramen; ma, mastoid process; mx, maxilla; n, nasal; nc, nuchal crest; p, parietal; pa, palatine; pmx, premaxilla; pnp, prenarial process; pop, postorbital process; sc, sagittal crest; sq, squamosal; zps, zygomatic process of squamosal.

**Figure 4:**
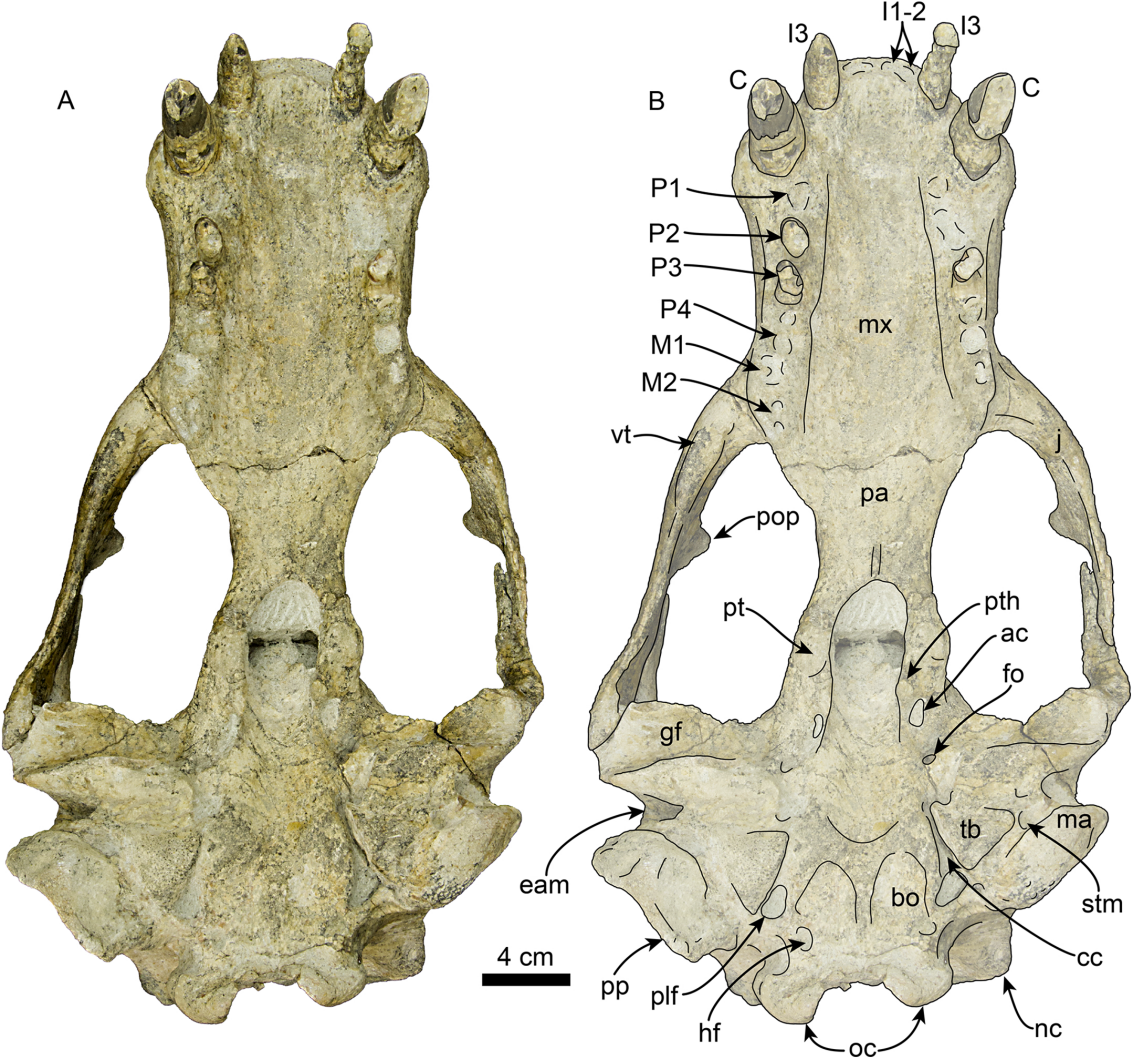
Skull of *Titanotaria orangensis* n. gen. et sp. (OCPC 11141). Skull in ventral view, without labels (A) and labelled (B). Abbreviations: ac, alisphenoid canal; bo, basioccipital; C, upper canine; cc, carotid canal; eam, external auditory meatus; fo, foramen ovale; gf, glenoid fossa; hf, hypoglossal foramen; j, jugal; I1–3, upper incisors 1–3; M1–2, upper molars 1–2; ma, mastoid; mx, maxilla; nc, nuchal crest; oc, occipital condyles; P1–4, upper premolars 1–4; pa, palatine; plf, posterior lacerate foramen; pop, postorbital process; pp, paroccipital process; pt, pterygoid; pth, pterygoid hamulus; stm, stylomastoid foramen; tb, tympanic bulla; vt, ventral tuberosity.

**Figure 5:**
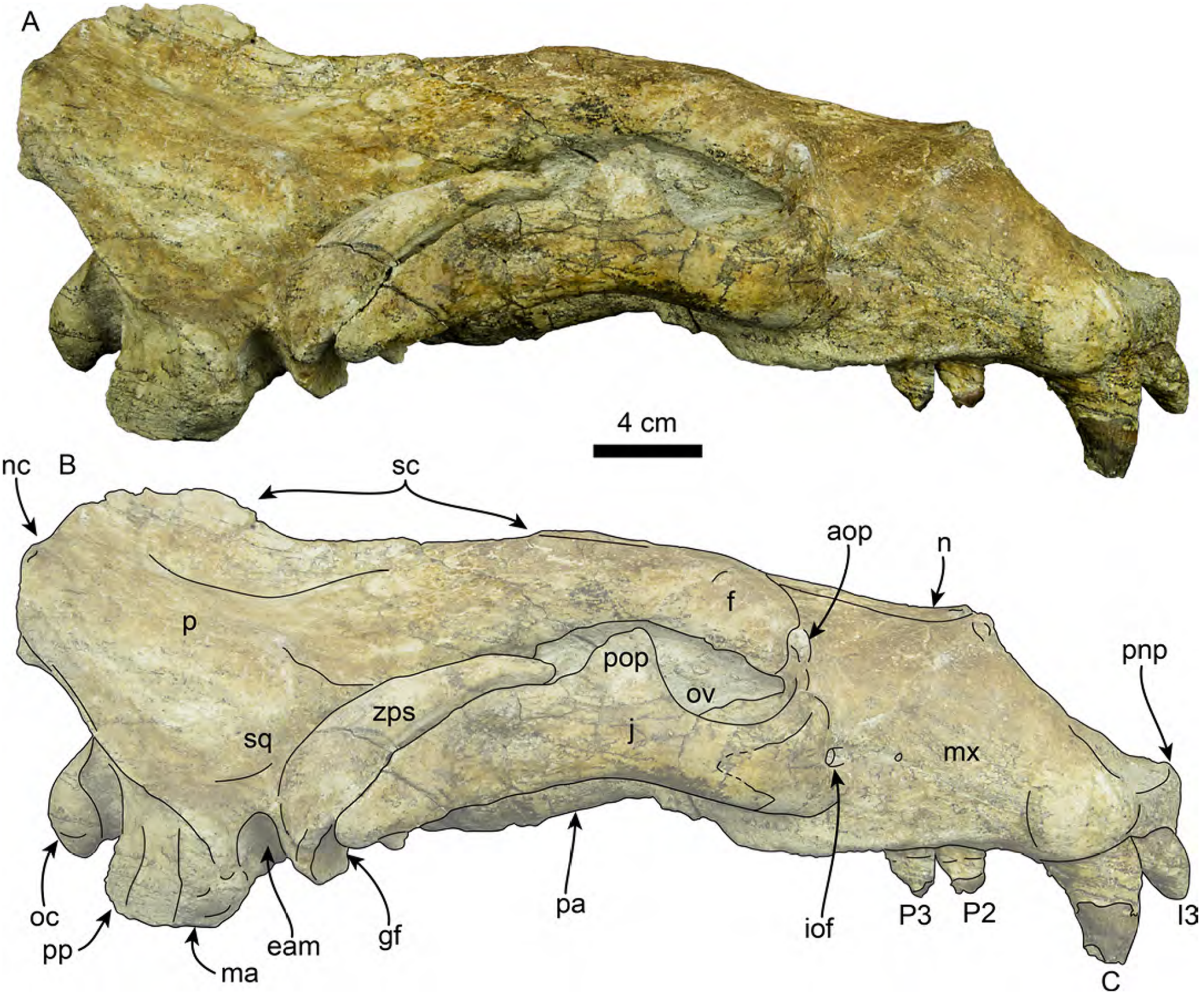
Skull of *Titanotaria orangensis* n. gen. et sp. (OCPC 11141). Skull in right lateral view, without labels (A) and labelled (B). Abbreviations: aop, antorbital process; C, upper canine; eam external auditory meatus; f, frontal; gf, glenoid fossa; I3, third upper incisor; iof, infraorbital foramen; j, jugal; ma, mastoid; mx, maxilla; n, nasal; nc, nuchal crest; oc, occipital condyle; ov, orbital vacuity; P2–3, upper premolars 2–3; p, parietal; pa, palatine; pnp, prenarial process; pop, postorbital process; pp, paroccipital process; sc, sagittal crest; sq, squamosal; zps, zygomatic process of squamosal.

**Figure 6:**
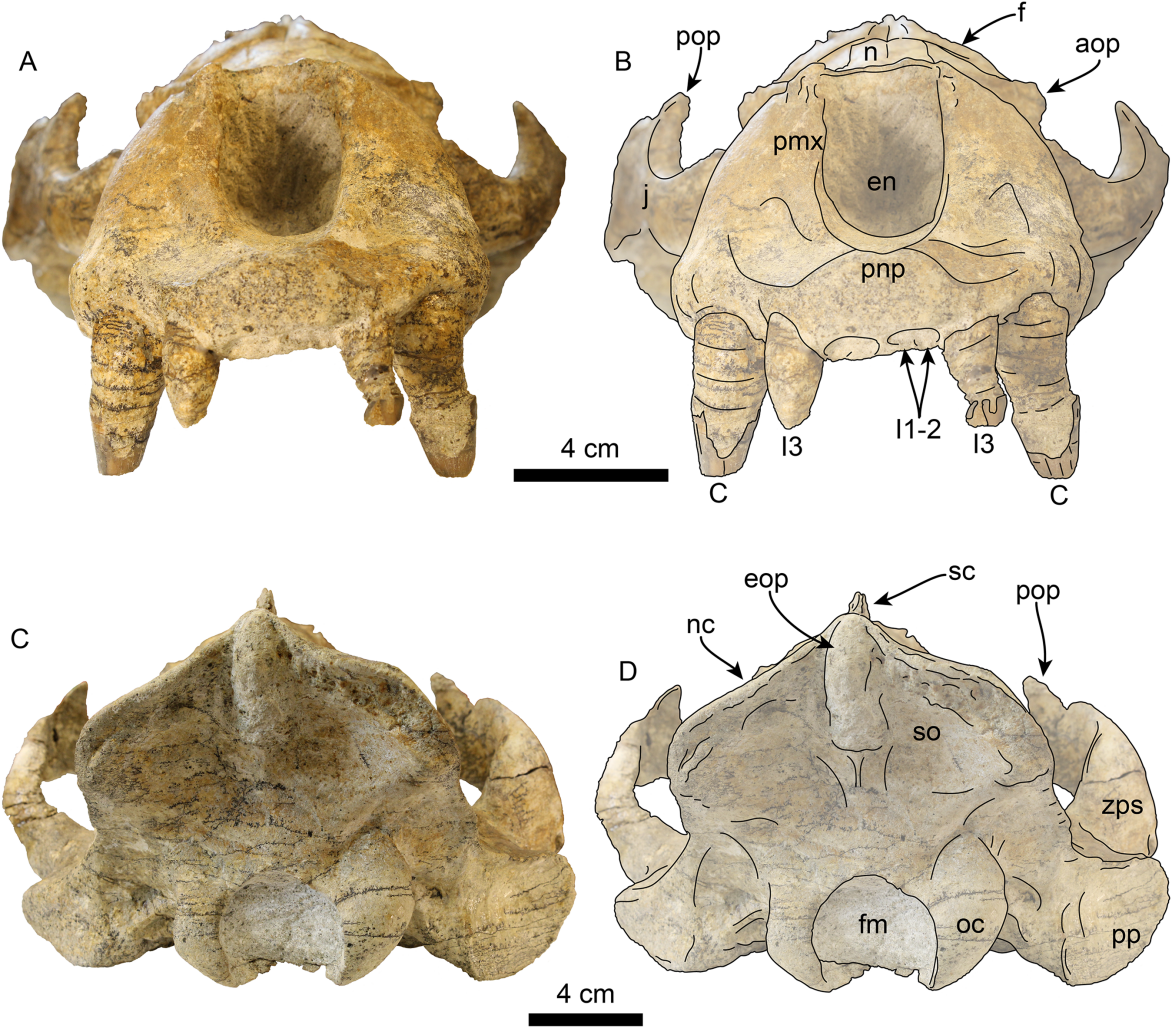
Skull of *Titanotaria orangensis* n. gen. et sp. (OCPC 11141). Skull in anterior (A–B), and posterior (C–D), views. Abbreviations: aop, antorbital process; C, upper canine; en, external nares; eop, external occipital protuberance; f, frontal; fm, foramen magnum; I1–3, upper incisors 1–3; j, jugal; n, nasal; nc, nuchal crest; oc, occipital condyle; pmx, premaxilla; pnp, prenarial process; pop, postorbital process; pp, paroccipital process; sc, sagittal crest; so, supraoccipital; zps, zygomatic process of squamosal.

**Figure 7:**
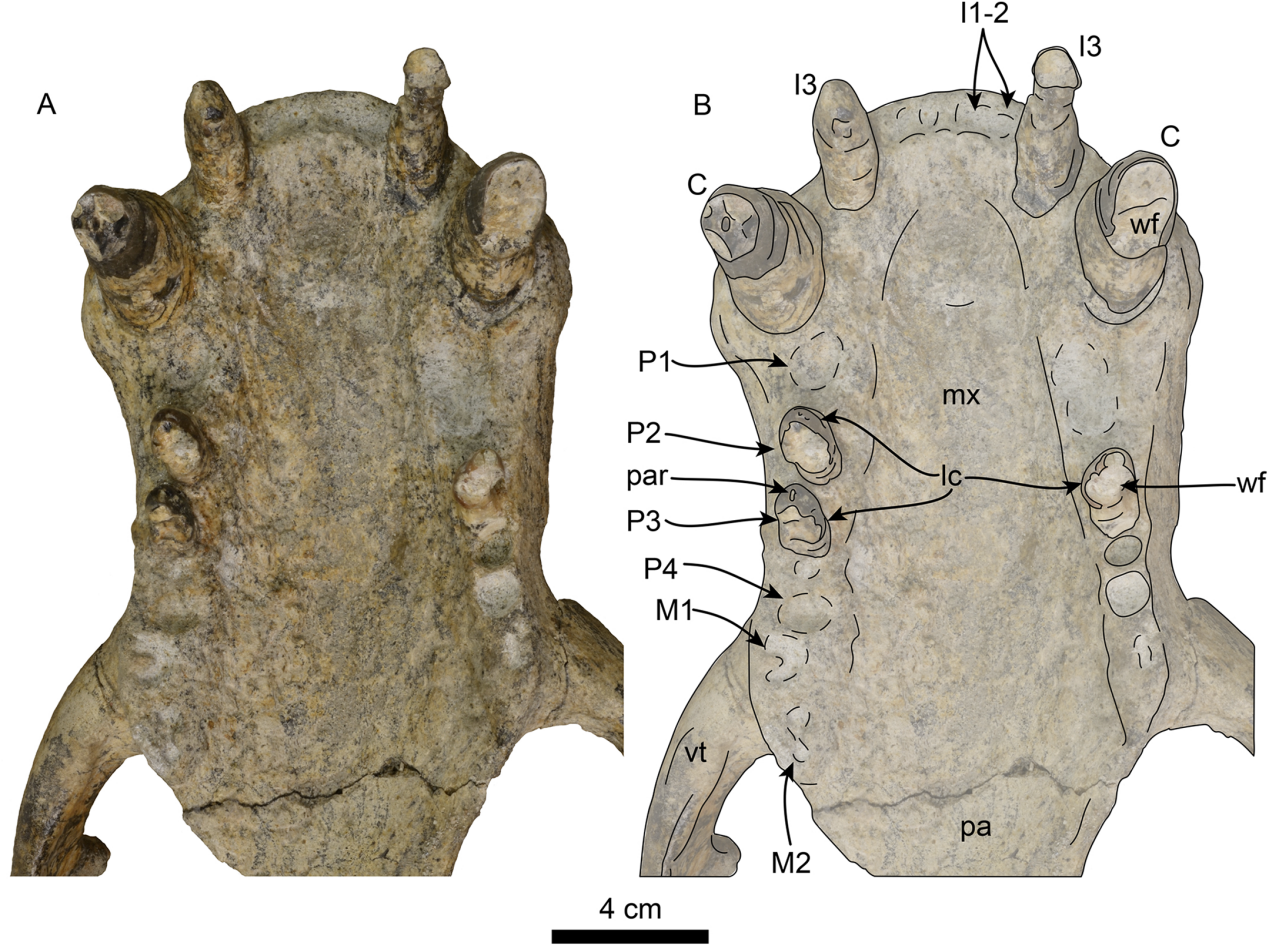
Skull of *Titanotaria orangensis* n. gen. et sp. (OCPC 11141). Detail of palatal region, without labels (A) and labelled (B). Abbreviations: C, upper canine; I1–3, upper incisors 1–3; lc, lingual cingulum; M1–2, upper molars 1–2; mx, maxilla; P1–4, upper premolars 1–4; pa, palatine; par, paracone; vt, ventral tuberosity; wf, wear facet.

The cranium (Figs. 3–7) is nearly complete and preserves diagnostic characters of the rostrum, frontal, parietal, zygomatic arches, palate, basicranium, and dentition. The skull also exhibits asymmetry, with the right side being more robust. This asymmetry is obvious when comparing the outline of the orbits and the relative dorsoventral thickness of the zygomatic arches. We attribute this asymmetry to a pathology on the left side of the skull, likely from a healed injury; a similar case was described in a modern pinniped by Howell (1925). Unless stated otherwise, the description of the cranium is based on the right side of the skull.

**Rostrum**—The rostrum (Figs. 3–7; Table 1) is anteroposteriorly elongate and expanded anterolaterally at about the level of the C1 roots. In lateral view, the premaxilla has a triangular outline, with its anterior end recurved and elevated slightly above the maxillary alveolar row. In dorsal view, the ascending processes of the premaxilla extend posteriorly creating short overlap between the nasal and maxilla (c. 3[1]). A prominent, knob-like, prenarial process is present on the anterodorsal tip of the premaxilla (c. 1[1]). The surface posterolateral to this process forms a shallow, oval concavity, dorsal to the space between the canine and incisors, and is interpreted as the area of origin for the m. lateralis nasi (Kastelein, Gerrits & Dubbeldam, 1991). The anterior narial opening is transversely compressed and dorsoventrally elliptical with thick lateral and ventral margins (c. 2[1]) (Fig. 3). Dorsally, the nasals are long (∼60% of the rostrum length), and their lateral edges are roughly parallel to each other with a posterior termination that forms a broad V-shaped frontal/nasal suture (c. 4[1]).

**Table 1:** Measurements (in mm) of skull of *Titanotaria orangensis* n. gen. et sp. (OCPC 11141).

Total length, premaxilla to intercondylar notch	403
Facial length	136.42
Orbit length	43, 57
Temporal fossa length	29, 42
C1–M2 toothrow length	L. (to shrunken M1): 127: R: 149
P1–M2 toothrow length	L. (to shrunken M1): 89 R: 113
External nares height	39
Transverse width of external nares	33
Length of nasals	94
Maximum transverse width of nasals	36
Transverse width of rostrum at C1	128
Narrowest width of rostrum	106
Transverse width of palate at M2	85
Bizygomatic width	238
Transverse width at mastoid	220
Transverse width across tympanic bullae	142 (Note: L side does not reach edge of skull)
Transverse width at paroccipital process	143
18. Transverse width of condyles	87
19. Height of foramen magnum	38
20. Width of foramen magnum	44
Anteroposterior length, mastoid, and paroccipital process	43, 55
Width of choanae between pterygoid hamuli	30
Infraorbital foramen, height/transverse width	L: 18/20; R: 11/14
Least interorbital width	45
Interorbital width at supraorbital process	No supraorbital process
Transverse width of braincase	142
Anteroposterior length of sagittal crest	140 or 199 depending on where the anterior edge of the crest is
Greatest depth of zygomatic arch	L: 45; R: 51
Height of occipital shield	93
C1 crown height/mesiodistal length/buccolingual width	L: 25*/20/20, R: 24*/23/20
P1 crown height/mesiodistal length/buccolingual width	L: Over prepared, can’t see R: a 15/12
P2 crown height/mesiodistal length/buccolingual width	L: Over prepared, can’t see R: 7/19/12
P3 crown height/mesiodistal length/buccolingual width	L: 6*/15/14 R: 13/16/12
P4 crown height/mesiodistal length/buccolingual width	L: a 24/12 R: a 20/15
M1 crown height/mesiodistal length/buccolingual width	L: a 15/6 R: a 17/12
M2 crown height/mesiodistal length/buccolingual width	L: Not present R: a 17/7

**Notes:**

Modified from Boessenecker & Churchill (2018).

a, alveoli; L, left; R, right.

* Broken, measured as preserved.

The maxilla is well preserved in dorsal and lateral aspect. In lateral view, the maxillary alveolar row is sinuous and swollen near the roots of the canines. The maxilla forms the anterior portion of the orbital wall and lacks a fossa muscularis (c. 14[1]); together with the frontal, the maxilla forms the antorbital process (c. 15[1]). In ventral view, the palatal margins of the maxilla are nearly parallel anteriorly, becoming more sinuous at the level of m1–2 (c. 10[0]). The palate is widest near the canines and transversely arched (c. 9[1]). The palatal width index (70) is greater than that of *Proneotherium repenningi* Barnes in Kohno, Barnes & Hirota 1995 (Deméré & Berta, 2001: 52–60), owing to its more parallel toothrows. The transverse palatal arch index of *T. orangensis* is greater than that of *P. repenningi* and *N. mirum*, becoming higher between P3–4 and again smaller at the level of M1–2 (Table 2; Deméré & Berta, 2001). The incisive foramina are largely obscured by sediment so their morphology is not discernible, but are located at about the level of the posterior half of the canines (c. 5[?]). The maxillo-palatine suture is fused; the palatines are elongated, fused along the midline, and posterolaterally expanded into the orbital region (c. 11[1]); their posterior margin is rounded.

**Table 2:** Measurements (in mm) used for palate width index and transverse palatal arch indices for *Titanotaria orangensis*, gen et sp. nov. OCPC 11141.

	C1 palate width	M1 palate width	Palate width index
	61	87	70
Tooth position	Transverse palatal depth	Transverse palatal width	Transverse palatal arch index
C1	12	61	19
P1	12	53	22
P2	14	60	23
P3	24	67	35
P4	26	72	36
M1	26	87	29
M2	22	84	26

**Note:**

Based on Deméré & Berta (2001).

**Zygomatic Arch**—The zygomatic arch is dorsoventrally broad, (Figs. 3–6) with a low, oval ventral tuberosity on the maxillary root (c. 7[1]) (Fig. 4). The dorsal and ventral struts of the maxillary root give the infraorbital foramen an elliptical outline. The infraorbital foramen is relatively small (c. 6[0]) with a maximum diameter of ∼14 mm, which is about the same diameter as in the much smaller skulls referred to *I. downsi* (USNM 23858 and USNM 184060; Repenning & Tedford, 1977). The jugal is dorsoventrally thick and has a relatively small triangular postorbital process (c. 18[0]), similar to that of *D. seftoni* and unlike the much larger process of *Valenictus chulavistensis* Deméré, 1994a. Anteriorly, the jugal-maxilla articulation is largely fused with the maxilla with an anterodorsal and anteroventral process, the latter not approaching the toothrow (c. 8[0]). Posteriorly, the zygomatic process of the jugal extends to the level just anterior to the preglenoid process on the right side, while is much shorter on the left (100 vs. 59 mm long). The zygomatic process of the squamosal is dorsally curved, relatively short and dorsoventrally thin relative to the jugal (c. 19[1]). The preglenoid process is relatively long, and the glenoid fossa is mediolaterally broad, deep, and is oriented anteroventrally (c. 26[0]). The squamosal fossa on the dorsal surface of the zygomatic root is relatively broad relative to the proportionately narrow fossa of *I. downsi* (e.g., USNM 184060), slopes anteroventrally (c. 25 [0]), and there is no indication of the presence of a transverse ridge (c. 27[0]).

**Frontal**—In dorsal view (Fig. 3), the interorbital bar is transversely narrow with parallel lateral margins that diverge anteriorly making the frontal widest anteriorly (c. 17[1]). Anteriorly, the frontals have a transversely concavo-convex contact with the maxilla, that continues along the lateral surface of the skull toward the antorbital region. The frontal lacks a supraorbital process (c. 16[1]). The temporal crests arise just posterior to the contact with the nasals, these then merge posteriorly form the anterior portion of the sagittal crest. In the orbito-temporal region the ventral margin of the frontal is dorsally convex and contacts the maxilla anteriorly and parietal posteriorly.

**Orbit**—The orbit (Figs. 3, 5 and 6) has an anterodorsal orbital wall that preserves a frontal-maxillary suture that bisects the antorbital process (c. 15[1]). A low ridge extends posteriorly from the antorbital process, forming the ventral edge of the orbit. A sediment filled orbital vacuity is located anteriorly in the orbital wall (c. 20[1]). The posterior margin of the orbital wall is formed by the orbitosphenoid and frontal. The orbitosphenoid is plate-like and extends anteriorly forming part of the optic foramen (c. 21[0]). The ventral half of the orbito-temporal region is dorsally convex and continues posteriorly to the pterygoid strut. The pterygoid strut is dorsoventrally thickened and transversely broad (c. 13[2]; Fig. 4). The hamular process of the pterygoid is small, transversely compressed, anteroposteriorly elongated, and projects posteroventrally (c. 12[0]).

**Parietal**—The parietal (Figs. 3 and 5) is fused with the frontals anteriorly and the squamosals laterally. The temporal surface is smoothly convex, with no visible pseudosylvian sulcus (c, 28[1]). In dorsal view, the parietals form most of the broad, boxlike braincase, which is roughly as wide transversely as long anteroposteriorly. The sagittal crest is prominent and elongate, extending from the frontal anteriorly and connecting with the nuchal crest posteriorly. The sagittal crest has a sinuous profile in lateral view, unlike the low posterodorsally sloping crest seen in other odobenids (e.g., *I. downsi*, *N. mirum*; Repenning & Tedford, 1977; Kohno, Barnes & Hirota, 1995). The nuchal crest is transversely wide and projects posterodorsally obscuring the occipital condyles (c. 32[0]). From the dorsal midline, the nuchal crest curves posterolaterally, then anteroventrally, reaching the mastoid process.

**Basicranium**—The basioccipital (Fig. 4) is broad and pentagonal in ventral view with anteriorly converging lateral margins and a prominent medial margin (c. 30[1]). The hypoglossal foramen is rounded and located anterior to the occipital condyle. The posterior lacerate foramen is rounded and located anterolateral to the hypoglossal foramen (c. 31[0]); anteromedially it merges with the carotid canal. The carotid canal is partially underlapped anteromedially by the tympanic bulla; both anterior and posterior openings are of similar diameter (∼9 mm) (c. 24[0]). The hyoid fossa is shallow (<10 mm) and relatively small, facing mainly posteroventrally; it is separated from the smaller (∼6 mm) stylomastoid foramen by a low ridge. The mastoid processes are large and broad (c. 33[1]), in contrast to the more delicate processes of *N. mirum* and early pinnipeds, while the paroccipital process is plate-like, posteriorly directed, and laterally continuous with the mastoid process (c. 34[3]). The tympanic bullae are well preserved, their ventral surface has a triangular outline and their ventral surface is shallowly convex, without any ornamentation. The ecto and entotympanic portions are indistinguishable from each other. There is no distinct posterior bullar process, as in the type of *I. downsi* (Mitchell, 1968). The stylomastoid foramen opens anterolaterally. The foramen ovale and alisphenoid canal are relatively small (∼14 mm diameter).

**Occipital region**—Posteriorly, the occipital surface is broadly concave, with a pentagonal outline. A thick external occipital protuberance extends ventrally from the convergence of the sagittal and nuchal crests to about 45 mm dorsal to the foramen magnum. The occipital condyles are large, robust, and form the margins of the circular foramen magnum. As with most of the skull, the occipital condyles are asymmetric, with the left condyle being noticeably smaller, and less prominent than the right one.

### Mandible and dentition

**Figure 8:**
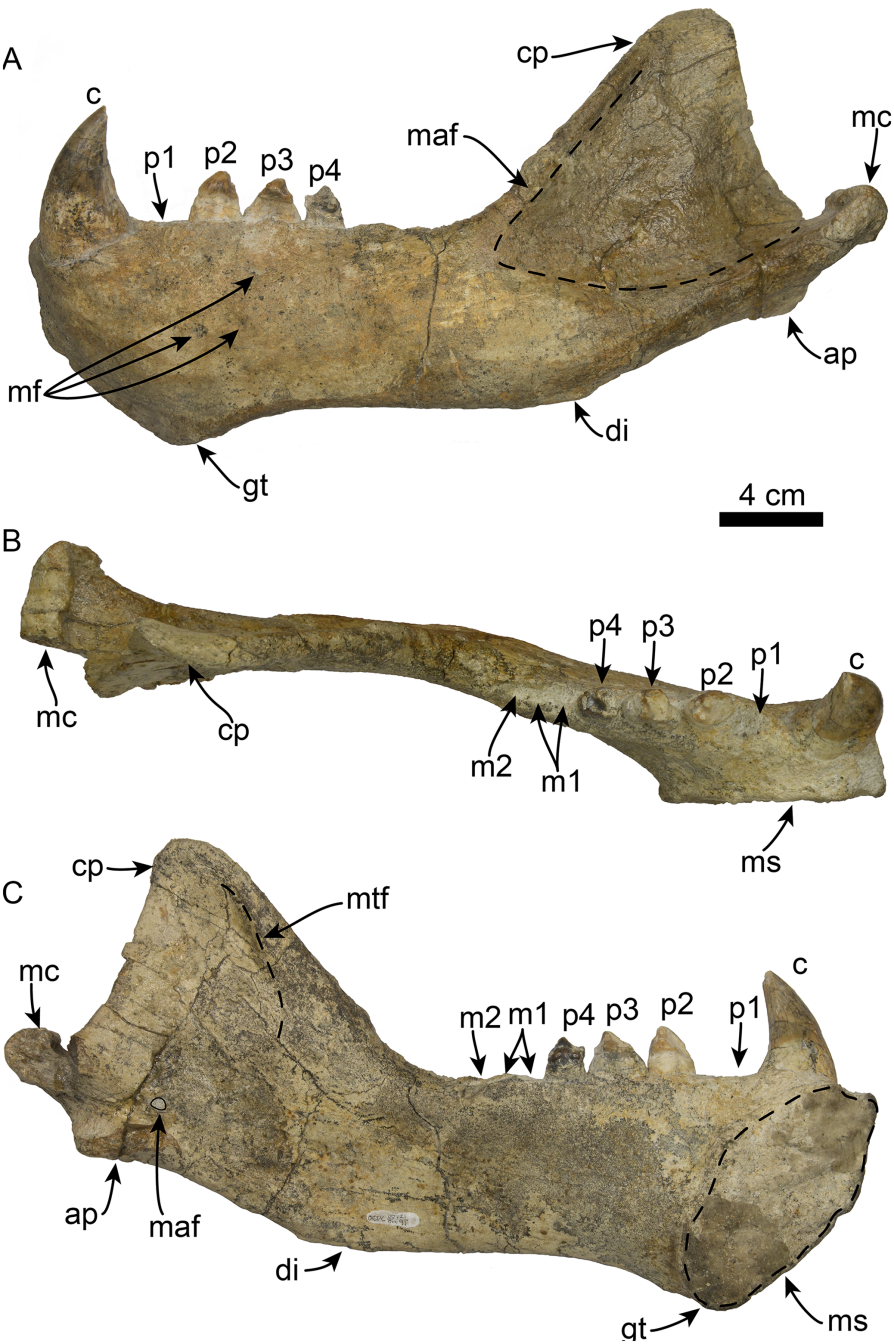
Mandible of *Titanotaria orangensis* n. gen. et sp. (OCPC 11141). Left mandible in lateral (A), occlusal (B), and medial (C), views. Abbreviations: ap, angular process; c, lower canine; cp, coronoid process; di, digastric insertion; gt, genial tuberosity; m1–2, lower molars 1–2; maf, masseteric fossa; mc, mandibular condyle; maf, mandibular foramen; mf, mental foramina; ms, mandibular symphysis; mtf, mandibular temporal fossa; p1–4, lower premolars 1–4.

**Figure 9:**
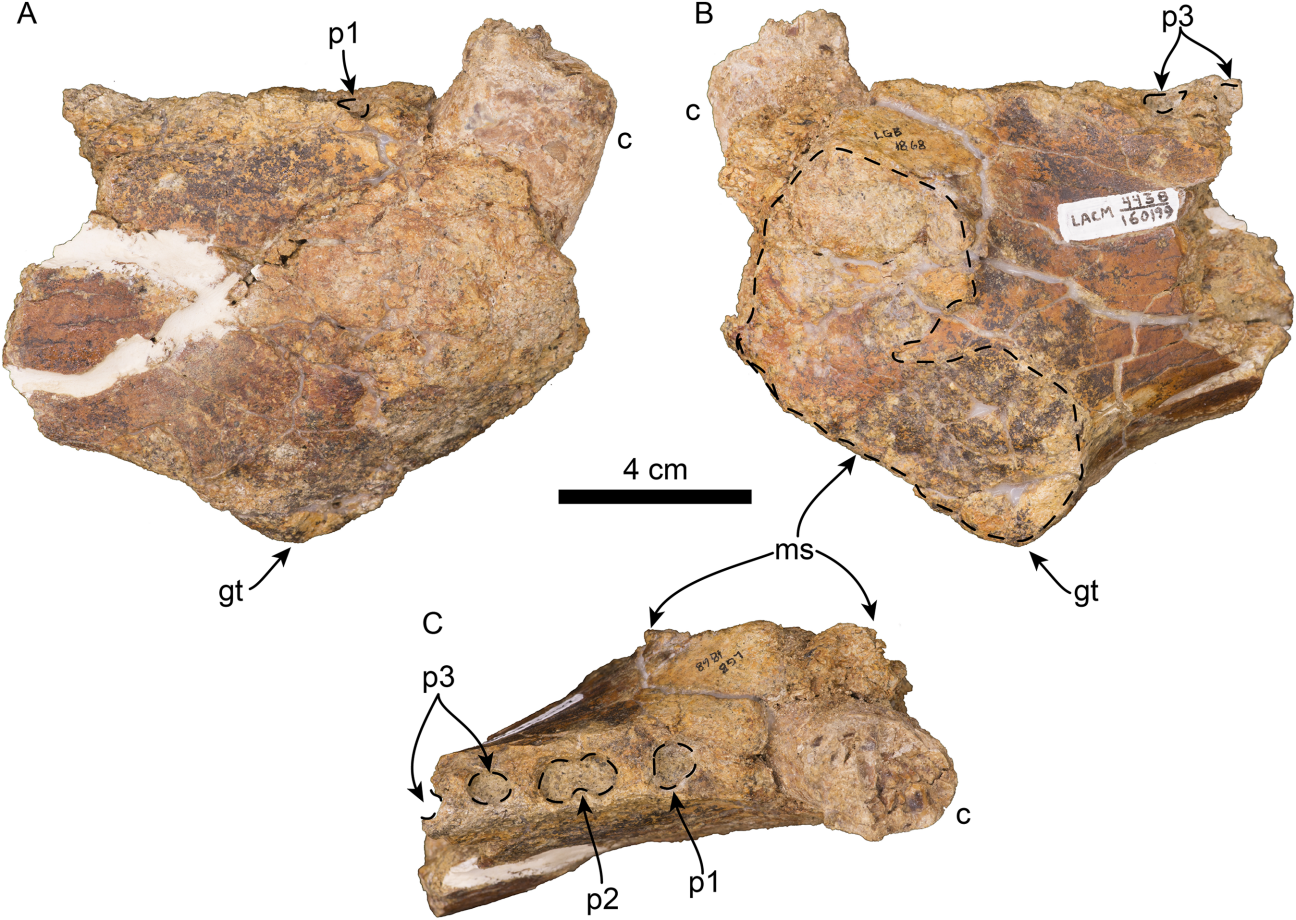
Referred partial mandible of *Titanotaria orangensis* n. gen. et sp. (LACM 160199). Left mandible in lateral (A), medial (B), and occlusal (C), views. Abbreviations: c, lower canine; gt, genial tuberosity; p1–3, lower premolars 1–3; ms, mandibular symphysis.

OCPC 11141 preserves both left and right mandibles while LACM 160199 is the anterior part of a right mandible (Figs. 8 and 9). In dorsal view, the articulated mandibles form a nearly parallel arch (c. 44[0]) and lack a mandibular furrow and edentulous mandibular terminus (c. 40[0], 41[0]). The right mandible lacks preserved dentition with the exception of a fragment of the canine. The left mandible preserves the canine as well as p2–4. The horizontal ramus has a straight dorsal and a moderately sinuous ventral margins (c. 39[0], 45[1]). The genial tuberosity is located at the posteroventral margin of the symphysis at a level between p1–2, and extends well below the ventral margin of the horizontal ramus forming the deepest part of the horizontal ramus (c. 37[2], 42[0], 43[1]). The mandibular symphysis is unfused with a rugose symphyseal surface, and occupies less than 50% of the length of the horizontal ramus (c. 35[0], 36[0]). The anterior surface of the symphysis is smooth, and curves anterodorsally from the genial tuberosity (c. 38[0], 40[0]). Several small, anteriorly oriented, mental foramina are present between p1–3 and the ventral border of the ramus. The anterior margin of the mandibular foramen is directed anteroventrally (c. 48[0]).

The coronoid process has a triangular outline and is anteroposteriorly narrow relative to the length of the mandible (c. 50[0]), its anterior edge ascends posterodorsally at about 52° from the horizontal ramus. The masseteric fossa is shallow, as in *I. downsi* (USNM 23858), and extends to a point near the anterior end of the coronoid process. Medially, the coronoid process has a shallow mandibular temporal fossa. The mandibular condyle is elevated only slightly above the level of the tooth row (c. 47[0]). The digastric insertion is moderately enlarged but not to the degree observed in *P. magnus* (c. 46[0]; Boessenecker & Churchill, 2013). The angular process is reduced, forming a small medial shelf (c. 49[1]).

**Upper dentition**—The preserved upper dentition (Fig. 7) and alveoli indicate a dental formula of I1–3, C1, P1–4, M1–2. The teeth preserved include left and right I3, C1 and P3, and right P2; while the rest of the teeth are represented by their respective alveoli. The alveolar morphology of I1–2 indicate anteroventrally directed single-rooted teeth. Both left and right I3 are present, but heavily worn, they are single-rooted, long and slender (c. 51[0], 52[0]). In occlusal view, I3 are laterally compressed and elliptical in cross section. The left I3 retains a small portion of the crown on its mesial side that begins 25 mm below the alveolar margin and has a distal wear facet; the crown of the right I3 is completely worn.

The canines are conical, ventrally directed, robust, and proportionate in size with respect to c1 (c. 55[0]). The base of the crown is oval in cross section. The enamel is smooth, and thick; the distal wear facet is large and elliptical on the left canine, and relatively smaller on the right.

Only the right P2 and left and right P3 are preserved. The alveolar morphology of P1 indicates a single-rooted tooth. P2 has a single, bi-lobed root, (c. 72[0]) and the crown is bulbous, and heavily worn on its distal surface. The paracone is partially preserved but extremely worn and broken at the apex; the occlusal wear has obliterated all other cusps. P2 also preserves a reduced crenulated lingual cingulum and lacks a labial cingulum (c. 71[0]).

P3 is double-rooted with cylindrical anterior and posterior root lobes that are of equal width as the crowns (c. 64[0], 74[0]). The crowns on both left and right P3 are poorly preserved, however, the right P3 preserves relatively more enamel with visible cusps. As in P2, the crown bulbous and the paracone forms the apex of the crown. Tooth wear on the distal surface has obliterated all other cusps. The lingual cingulum is partially worn, but it still preserves some of the crenations; there is no labial cingulum.

Although P4 and M1 are missing, the alveolar morphology indicates they were double-rooted teeth with cylindrical anterior and posterior root lobes (c. 76[1], 77[1]). M2 was apparently much smaller than M1; the preserved alveoli indicate it was a double-rooted tooth (c. 80[0]).

**Figure 10:**
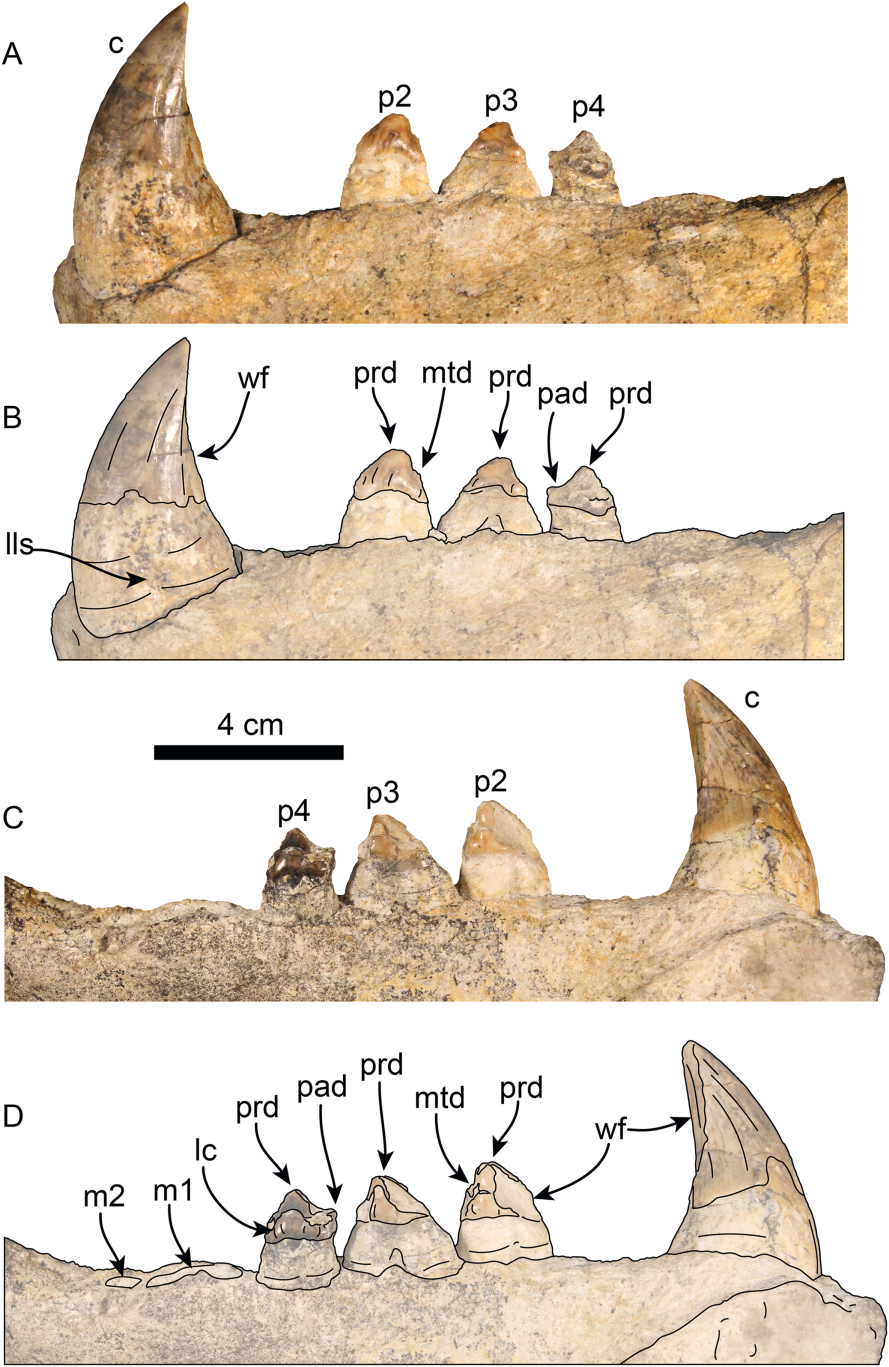
Lower dentition of *Titanotaria orangensis* n. gen. et sp. (OCPC 11141). Left lower dentition in labial (A–B) and lingual (C–D), views. Abbreviations: c, lower canine; lc, lingual cingulum; lls, lingual longitudinal sulcus; m1–2, lower molars 1–2; mtd, metaconid; p2–4, lower premolars 2–4; pad, paraconid; prd, protoconid; wf, wear facet.

**Lower dentition**—The left and right mandibles of OCPC 11141 (Fig. 10; Table 3) preserve c1 and p2–4, and c1, respectively; alveoli for p1, m1–2, for a tooth count of i?, c, p1–4, m1–2 (c. 53[?], 61[0]). The tooth row is relatively short making up less than 40% of the mandible length (c. 65[1]). The crowns have well-developed enamel (c. 62[0]), with wear surfaces on the apex and distal and mesial surfaces (c. 82[1]). The incisors are missing in both mandibles, and their alveolar morphology is obscured by sediment.

**Table 3:** Measurements (in mm) of mandible of *Titanotaria orangensis* n. gen. et sp. (OCPC 11141).

Total length	231, 247
Length of toothrow, c1–m2	140, 146
Height at genial tuberosity	86, 82
Greatest length of symphysis	62
Greatest height at coronoid process	121, 115
Transverse width of mandibular condyle	38*, 58
Length of masseteric fossa	∼106, ∼109 (Not distinct)
Length of coronoid	118, 118
c1 crown height/mesiodistal length/buccolingual width	33*/24/19, n/a
p1 crown height/mesiodistal length/buccolingual width	L: Alveolus poorly preserved R: 15/12
p2 crown height/mesiodistal length/buccolingual width	L: 9/15/12 R: Alveolus poorly preserved
p3 crown height/mesiodistal length/buccolingual width	L: 9/13/1 R: Alveolus poorly preserved
p4 crown height/mesiodistal length/buccolingual width	L: 11*/14/11 R: Alveolus poorly preserved
m1 crown height/mesiodistal length/buccolingual width	Alveoli poorly preserved
m2 crown height/mesiodistal length/buccolingual width	Alveoli poorly preserved

**Notes:**

Modified from Boessenecker & Churchill (2018).

a, alveoli; L, left; R, right.

* Broken, measured as preserved.

The lower canines are nearly as large as the upper ones, vertically oriented, and posteriorly curved (c. 56[0], 60[0]). The crown is conical, transversely compressed, with an oval cross section; the enamel is smooth and thick with a posterior crista (c. 57[0], 58[0]), and posterior wear facet formed by occlusion with the upper canines. The lingual and labial surfaces of the canine roots exhibits longitudinal sulci that terminate below the base of the crown, giving it a bilobed cross section (c. 59[1]) as in *I. downsi*, *P. thomasi*, and *N. mirum* (Mitchell, 1968; Barnes, 1988; Velez-Juarbe, in press).

The first premolar (p1) is missing on the type and referred mandibles, however, the alveoli indicate a single, cylindrical root. The second through fourth premolars (p2–4) are double-rooted or bilobed. Their crowns are bulbous with oval outline (c. 63[1]) and are worn on their mesial and distal surfaces. Premolar 1 has a prominent protoconid cusp; mesiolingual wear preclude us from determining if there were other cusps (c. 68[?]); distally, the crown is worn but there are remnants of a small metaconid (c. 70[0]). Lingually, a crenulated cingulum is present (c. 67[1]), while distolabially there is an incipient cingulum.

The left p3 is double-rooted with divergent and bulbous anterior and posterior root lobes that result in a root as wide as the crown (Character 64[0], 73[0]). The crown is bulbous and as in p2, the crown has a prominent protoconid cusp, the wear facets have nearly obliterated other cusps. The lingual cingulum is worn mesially and distally, but it was prominent with small crenulations.

The left p4 resembles p3 and is double-rooted with cylindrical anterior and posterior root lobes, but which are not as wide as the crown. Although the crown is damaged on its labial side, the lingual surface is well preserved; the crown is bulbous with small mesial and distal wear facets; with a prominent, crenulated lingual cingulum that borders a small talonid basin (c. 69[1]), prominent protoconid and paraconid cusps [c. 66 [0]).

The molars are not preserved in any of the mandibles. But, the alveolar morphology suggests m1 has double-rooted cylindrical roots while m2 had a single, oval root (c. 78[0], 79[0], 81[0]).

### Postcrania

**Figure 11:**
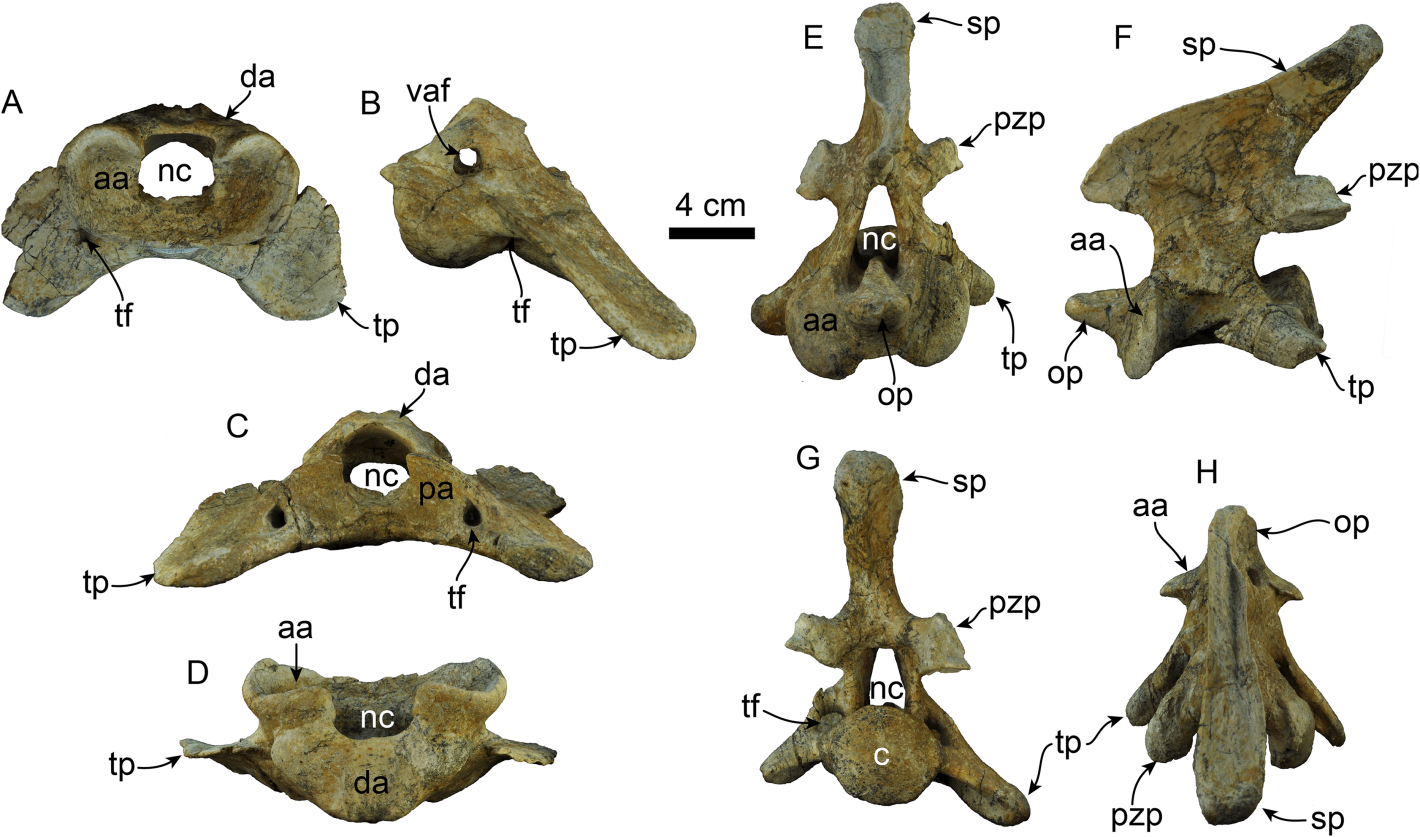
Atlas and axis of *Titanotaria orangensis* n. gen. et sp. (OCPC 11141). Atlas in anterior (A), left lateral (B), posterior (C), and dorsal (D) views; axis in anterior (E), left lateral (F), posterior (G), and dorsal (H) views. Abbreviations: aa, anterior articular surface; c, centrum; da, dorsal arch; nc, neural canal; op, odontoid process; pa, posterior articular surface; pzp, postzygapophysis; sp, spinous process; tf, transverse foramen; tp, transverse process; vaf, vertebrarterial foramen.

**Figure 12:**
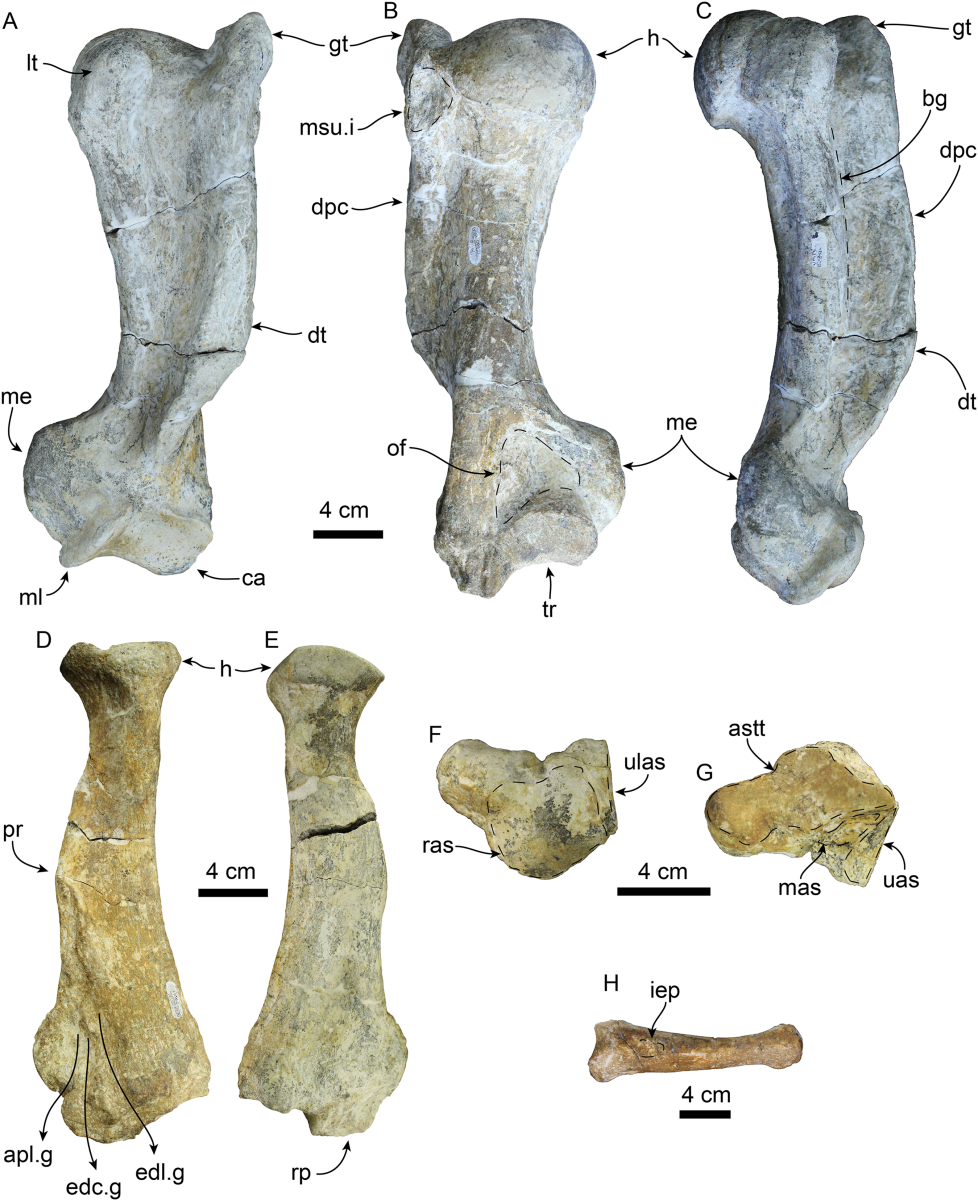
Forelimb elements of *Titanotaria orangensis* n. gen. et sp. (OCPC 11141). Left humerus in anterior (A), posterior (B), and medial (C) views; left radius in lateral (D), and medial (E), views; left scapholunar in proximal (F), and dorsodistal (G), views; left metacarpal I in dorsal (H) view. Abbreviations: apl.g, groove for tendon of M. abductor policis; astt, articular surface for trapezium and trapezoid; bg, bicipital groove; ca, capitulum; dpc, deltopectoral crest; dt, deltoid tubercle; edc.g, groove for tendon of M. extensor digitorum communis; edl.g, groove for tendon of M. extensor digitorum lateralis; gt, greater tubercle; h, head; iep, insertion for M. extensor pollicis; lt, lesser tubercle; mas, magnum articular surface; me, medial epicodyle; ml, medial lip of trochlea; msu.i, insertion of M. supraspinatus; of, olecranon fossa; pr, pronator ridge; ras, radial articular surface; rp, radial process; tr, trochlea; uas, unciform articular surface; ulas, ulnar articular surface.

The holotype includes an associated, mostly complete postcranial skeleton. However, here we describe the baculum, atlas, and phylogenetically informative postcranial elements (Figs. 10–12; Tables 4–7), which include the atlas, axis, humerus, radius, scapholunar, metacarpal 1, astragalus, calcaneum, and entocuneiform.

**Table 4:** Measurements (in mm) of postcranial elements of *Titanotaria orangensis* n. gen. et sp. (OCPC 11141), excluding the humerus and radius (shown separately).

**Atlas**	
Width across anterior articular surface	111
Maximum height along midline	78
Maximum width across transverse processes	191+
Height/width of neural canal anteriorly	36/36
Diameter of transverse foramen	10
Width across posterior articular surface	79
**Axis**	
Width across anterior articular surfaces	75
Maximum height along midline	152
Height of neural spine	84
Height/width of neural canal anteriorly	32/18
Maximum width across postzygapophyses	79
Maximum width across transverse processes	117
Maximum diameter of transverse foramina	17
Maximum length	112
Maximum length of neural spine	140
Height/width of centrum posteriorly	43.54
**Scapholunar**	
Maximum length	80
Maximum width	64
**Metacarpal I**	
Maximum length	160
Maximum width/height proximally	44/31
Maximum width/height distally1	33/26
**Astragalus**	
Maximum length	95
Maximum width	76
Transverse width of capitulum	58
Maximum height of capitulum	34
Width of neck	45
**Calcaneus**	
Maximum length	106
Maximum distal width	75
Maximum proximal width	59
**Entocuneiform**	
Width/height of metatarsal I articular surface	47/23
Maximum width/length dorsally	54/45
Width/height of navicular facet	30/25
Width/height of mesocuneiform facet	21/9
**Baculum**	
Maximum length	342
Proximal diameter	34
Maximum height of distal end	28
Mid-shaft diameter	24

**Notes:**

Modified from Boessenecker & Churchill (2018).

+, measured on incomplete surface.

**Atlas**—The atlas (Figs. 11A–11D) is virtually complete, missing only a fragment of the right transverse process. The anterior articular surface is shallowly concave transversely and dorsoventrally, forming a nearly continuous surface ventrally. The dorsal arch has a low midline crest dorsally. The neural canal is round in outline. The vertebrarterial foramina are round (diam. ∼10 mm) and open laterally. The transverse processes are arcuate, flange-like and oriented posteroventrally. The transverse foramina open anteriorly within a shallow fossa at the base of the transverse processes; posteriorly they open anteromedially on the transverse process. The posterior articular surfaces are shallowly concave dorsoventrally and are smaller than their anterior counterparts. The articular surface for the odontoid process is shallowly convex and faces dorsally.

**Axis**—The axis (Figs. 11E–11H) is nearly complete, missing only the distal end of the left transverse process. The odontoid process is cylindrical and forms a continuous surface with the anterior articular surfaces, which are shallowly convex. Proximally, the dorsal surface of the odontoid forms a low keel, which continues posteriorly into the floor of the neural canal. The neural canal is oval to subtriangular in outline. The transverse processes are oriented posteroventrally and are pierced proximally by the transverse foramina. The postzygapophyses are oriented posteriorly with relatively large mammilary processes. The neural spine is tall and slopes posterodorsally with a blade-like outline in lateral view (c. 91[1]), similar to the condition of *A. akamatsui* (Tanaka & Kohno, 2015). The dorsal surface of the neural spine is flat and transversely expanded. The posterior surface of the centrum is shallowly convex with a rounded outline.

**Figure 13:**
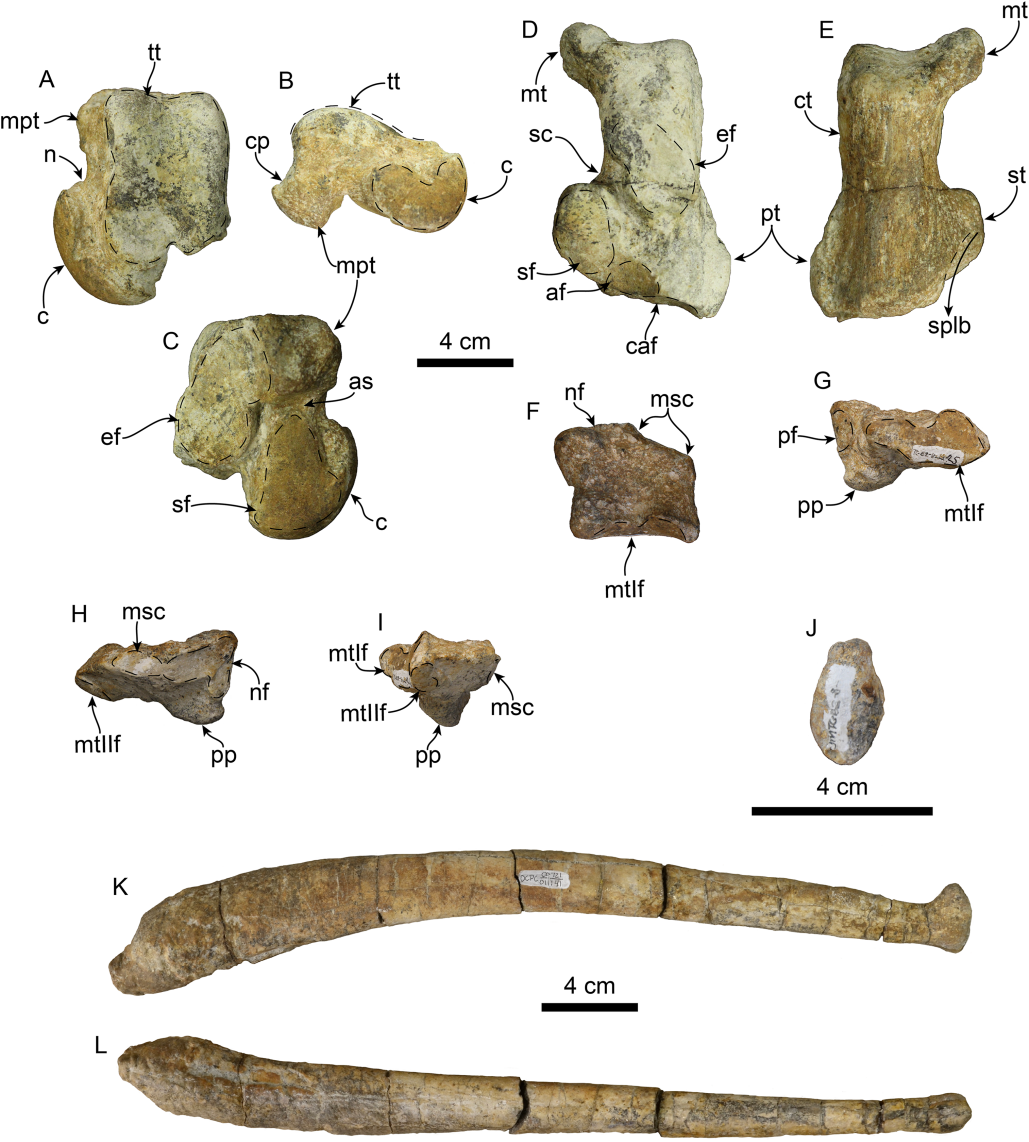
Hindlimb elements and baculum of *Titanotaria orangensis* n. gen. et sp. (OCPC 11141). Left astragualus in tibial (A), medial (B), and ventral (C) views; left calcaneum in dorsal (D), and plantar (E) views; left entocuneiform in dorsal (F), distal (G), proximal (H), and lateral (I), views; baculum in proximal (J), right lateral (I), and ventral (L) views. Abbreviations: af, accessory facet; astragalar sulcus; c, capitulum; caf, cuboid articular facet; cp, calcaneal process; ct, calcaneal tuber; ef, ectal facet; mpt, medial plantar tuberosity; msc, mesocuneiform facet; mt, medial tuberosity; mtIf, metatarsal I facet; n, neck; nf, navicular facet; pf, pisiform facet; pp, plantar process; pt, peroneal tubercle; sc, sulcus calcanei; sf, sustentacular facet; splb, sulcus for peroneus longus and peroneus brevis tendon; st, sustentaculum tali; tt, tibial trochlea.

**Baculum**—The baculum (Figs. 13J–13L) is nearly cylindrical, dorsally arched, and elliptical in cross-section. The ventral surface is nearly flat, while dorsally it is rounded. The proximal end is bulbous with a rough surface, while distally its diameter becomes smaller toward the distal end. The distal end is anteriorly convex and dorsoventrally expanded in the form of dorsal and ventral knobs. The baculum of *T. orangensis* is 10% of the estimated body length (34.2 cm/est body length 331 cm). This is considerably smaller than that of *O. rosmarus*, which has the relatively largest baculum among pinnipeds, with its length being equivalent to around 18% of the body length (Fay, 1982; Miller, 2018).

**Humerus**—The left humerus is laterally bowed (Figs. 12A–12C; Tables 5 and 6), and relatively robust, with length/least diameter and proximal width/greatest length ratios between those of *Valenictus* spp. (more robust/stocky) and those of the more slender *D. seftoni* and *I. downsi* (Repenning & Tedford, 1977; Deméré, 1994a; Table 6). The greater tuberosity is prominent extending proximally beyond the level of the head as in *I. downsi*, *G. pugnax*, and *D. seftoni* (Mitchell, 1968; Repenning & Tedford, 1977; Barnes & Raschke, 1991; Deméré, 1994a). The humeral head is large and rounded. The lesser tuberosity is knob-like and does not extend proximally beyond the level of the head, thus differing from the condition of *I. downsi* and being more similar to *D. seftoni* (Mitchell, 1968; Repenning & Tedford, 1977; Deméré, 1994a). In lateral or medial view the deltopectoral crest is moderately prominent, resembling the condition observed in *P. pacifica* and *Valenictus* spp., and differing from the more prominent crest of *I. downsi* (Mitchell, 1961; Repenning & Tedford, 1977; Deméré, 1994a). The deltopectoral crest gradually tapers distally approaching the radial fossa, and curves toward the medial lip of the trochlea. The deltoid tubercle is on the pectoral crest (c. 83[0]). The distal articular surface has a saddle-like outline in posterior or anterior views (Figs. 12A and 12B). The medial lip of the trochlea is prominent with a diameter greater than that of the capitulum (c. 84[1]). The coronoid and radial fossae are present, with the former being deeper and wider. The medial epicondyle is prominent, with an oval outline as in *I. downsi* and *D. seftoni*, and unlike the more prominent medial epicondyle that distinguishes *Valenictus* spp. (Mitchell, 1961, 1968; Repenning & Tedford, 1977; Deméré, 1994a).

**Table 5:** Measurements (in mm) of humerus of *Titanotaria orangensis* n. gen. et sp. (OCPC 11141).

Greatest length, tuberosity to radial capitulum	345
Length, head to radial capitulum	331
Length, lesser tuberosity to radial capitulum	324
Transverse width across tuberosities	116
Greatest transverse width of head	96
Transverse width at narrowest part of shaft	54
Anteroposterior width at midshaft	74
Greatest width across epicondyles	125
Greatest anteroposterior diameter of medial edge of trochlea	70
Greatest anteroposterior diameter of radial capitulum	43
Greatest width of distal articulation	83
Transverse width of entepicondyle	36

**Note:**

Modified from Deméré (1994a).

**Table 6:** Measurements (in mm) of odobenid humeri used for comparison of robustness.

		GL	LD	TW	GL/LD	TW/GL
*Titanotaria orangensis*	OCPC 11141	345	54	116	6.38	0.33
*Valenictus chulavistensis*	SDSNH 36786^1^	326	56	120	5.82	0.37
*Valenictus chulavistensis*	SDSNH 38312^1^	315	55	120	5.72	0.38
*Valenictus chulavistensis*	SDSNH 38315^1^	306	56	119	5.46	0.39
*Valenictus chulavistensis*	SDSNH 35375^1^	263	61	109	4.31	0.41
*Valenictus chulavistensis*	SDSNH 38300^1^	300	63	112	4.76	0.37
*Valenictus imperialensis*	LACM 3926^1^	253	51	102	4.96	0.40
*Dusignathus seftoni*	SDSNH 43873^1^	346	62	96	5.68	0.26
*Imagotaria downsi*	USNM 23870^2^	265	42	85	6.30	0.22
*Imagotaria downsi*	USNM 23865^2^	226	37	65	6.10	0.29

**Notes:**

GL, greatest length; LD, least diameter of shaft; TW, transverse width at tuberosities.

1 Measurements from Deméré (1994a);

2 Measurements from Repenning & Tedford (1977).

**Radius**—The radius (Figs. 12D and 12E) is stout with its distal end anteroposteriorly expanded with a large radial process (c. 85[2]); it is similar in size to one of the referred radii of *I. downsi* (Repenning & Tedford, 1977: table 9). Proximally, the head of the radius is rounded and with a shallowly concave articular surface. The proximal end of the diaphysis is transversely narrow relative to the distal end, and oval in cross section. The pronator ridge is prominent and located about midlength on the shaft. Distally, the radial crest is rounded in lateral view, and together with the radial process, it forms the widest part of the radius (Table 7). The radial process is not as displaced medially as that of *Imagotaria*. Distally, the anterolateral surface is marked by relatively deep grooves for the M. extensor metacarpi pollicis and M. extensor digitorum.

**Table 7:** Measurements (in mm) of radius of *Titanotaria orangensis* n. gen. et sp. (OCPC 11141).

Length	277
Greatest width, proximal articulation	73
Anteroposterior length, distal termination	95
Anteroposterior length, proximal articulation	54
Depth of shaft at pronator teres origin	58
Width of shaft at pronator teres origin	35

**Note:**

Modified from Repenning & Tedford (1977).

**Scapholunar**—Proximally (Fig. 11E), the radial articular surface is anteroposteriorly convex with a subrectangular outline. Distally (Fig. 11F), the articular surface for the trapezium and trapezoid is oval and deeply concave transversely. The unciform articular surface is elongated and shallowly concave and is continuous posteriorly with the cuneiform articular surface. The magnum articular surface is deeply concave, forming a distinct pocket (c. 87[1]) as in other odobenids (Repenning & Tedford, 1977; Deméré, 1994b).

**Metacarpal 1**—The first metacarpal (Fig. 11) is elongated, curved laterally, and slender, resembling that of *I. downsi* (Repenning & Tedford, 1977). The proximal surface of the bone is smoothly concave transversely and dorsoventrally convex. Proximally, the dorsal surface has a shallow oval depression for the insertion of M. extensor pollicis (c. 86[1]). The distal articular trochlea has a rounded outline.

**Astragalus**—The head of the astragalus (Fig. 12) is prominent and dorsoventrally flattened; the neck is well defined and the articular surface is dorsoventrally and transversely convex. Ventrally, the navicular facet is continuous with a broad, oval (longer than wide) sustentacular facet, markedly differing from the relatively narrow navicular facet of *Valenictus* (Deméré, 1994a; Boessenecker, 2017). The sustentacular facet is separated from the ectal facet by a narrow astragalar sulcus. The ectal facet is concave and lunate, and separated from the oval, fibular facet by a low ridge. Dorsally, the navicular facet bears a median notch, and is separated from the trochlea by a groove. The trochlea is rectangular in outline with a short, shallow intertrochlear pit along its distal edge; the articular surface is convex and divided by a shallow anteroposteriorly-oriented groove. The lateral trochlear ridge is longer than the medial trochlear ridge. The calcaneal process is short and rounded (c. 88[1]). The medial plantar tuberosity is rounded and more robust and prominent than the nearly inconspicuous lateral plantar tuberosity.

**Calcaneum**—The calcaneal tuber is elongated with a prominent medial process (= internal tuberosity of Repenning & Tedford (1977)) (c. 89[1]; Figs. 13D and 13E), as in most odobenids and *Allodesmus* spp. (Mitchell, 1966; Deméré, 1994b; Tonomori et al., 2018). The posterior articular surface is convex and broad. The sustentaculum tali is robust, extending as far medially as the medial process of the calcaneal tuber. The ectal face is anteroposteriorly convex, with an oval outline, and oriented obliquely to the long axis of the bone. The sustentacular facet is flat posteriorly, becoming concave distally; a secondary shelf in the sustentaculum is missing, as in *I. downsi* and *V. chulavistensis* (Repenning & Tedford, 1977; Deméré, 1994a). The groove for the peroneus longus tendon extends along the distoventral surface of the sustentaculum tali. Distally, the calcaneum is mediolaterally broader than proximally, similar to the condition in *I. downsi*, but unlike the proportionately broader distal end of the calcanei of *V. chulavistensis* (Repenning & Tedford, 1977; Deméré, 1994a). On the distal surface, the cuboid articular surface is broad, subrounded, and makes up most of the distal surface of the calcaneum.

**Entocuneiform**—The entocuneiform (Figs. 13F–13H) has an irregularly pentagonal outline, and is mediolaterally wide and dorsoventrally flattened (Table 4). In proximal (posterior) view the entocuneiform is divided into two facets. The medial facet for the navicular is shallowly convex, L-shaped and oriented proximally, while the mesocuneiform facet is oriented proximolaterally, both forming a broad angle (c. 90[1]). Proximally, the medial surface has a suboval pisiform facet. The anterolateral surface of the bone has a ventrolaterally oriented oval facet for metatarsal II. Ventrally, there is a rounded, prominent, plantar process on the proximomedial corner. Anteriorly, the articular surface for metatarsal I is irregularly oval, with a dorsomedial depression; the surface is transversely concave and dorsoventrally convex.

## Results

### Phylogenetic results

Our phylogenetic analysis without ordered characters recovered 27 equally parsimonious trees of 265 steps. An analysis with ordered characters, recovered 27 equally parsimonious trees of 274 steps (Fig. 13). Both analyses recover nine trees that vary within the ingroup (Odobenidae). In the strict consensus trees for both analyses, the topology for the ingroup is almost entirely pectinate except for three polytomies that can be resolved to form exclusive sister taxa: (1) A polytomy that includes *N. mirum*, *Kamtschatarctos sinelnikovae* Dubrovo, 1981, and odobenids more closely related to *O. rosmarus*. The shortest trees include *N. mirum* and *K. sinelnikovae* as sister taxa or paraphyletic with either taxon as more basal; (2) A polytomy that includes *D. seftoni*, *D. santacruzensis*, and odobenids more closely related to *O. rosmarus*. The shortest trees include a monophyletic *Dusignathus* Kellogg, 1927 and a paraphyletic *Dusignathus*, with *D. santacruzensis* as sister to the Odobeninae. In the unordered analysis, *G. pugnax* is always sister to the *Dusignathus* polytomy, but the shortest trees from the ordered analysis also include *G. pugnax* as more closely related to *O. rosmarus* than a monophyletic *Dusignathus*. (3) A polytomy that includes *P. japonicus*, *O. emmonsi*, and odobenids more closely related to *O. rosmarus*. The shortest trees include *P. japonicus* as either the sister taxon of *O. emmonsi* or the sister taxon of a clade that includes *O. emmonsi* and odobenids more closely related to *O. rosmarus. Titanotaria orangensis* is found to be sister to a clade that includes *I. downsi* and all other more crownward odobenids, albeit with low support. *Titanotaria orangensis*, *Pelagiarctos* sp., and *I. downsi* represent a grade of phenetically similar taxa at the base of the clade that includes *P. magnus* with *Dusignathus*, *G. pugnax*, and Odobeninae.

### Phylogenetic taxonomy

The first study to provide phylogenetic definitions for Odobenidae and its subclades was Deméré (1994b), which demonstrated the paraphyly of the “Imagotariinae” Mitchell, 1968, and provided phylogenetic definitions for Dusignathinae Mitchell, 1968, Odobenidae Allen, 1880, Odobeninae Mitchell, 1968, and the new taxon Odobenini Deméré, 1994b. Since that time, all workers have accepted the non-monophyly of the “Imagotariinae,” although this term is used as an informal grade by some authors (Boessenecker & Churchill, 2013). Given that *I. dowsni* is more closely related to *P. magnus* and other late Miocene odobenids, the continued referral of the most basal middle Miocene forms (with more plesiomorphic characters) as imagotariines is misleading. Some studies have chosen to refer to more basal forms as “archaic odobenids,” although the boundary of this informal grouping can differ even within a single paper (see Tanaka & Kohno, 2015: fig. 11 vs. text).

The definition of Dusignathinae Mitchell, 1968, proposed by Deméré (1994b) is more exclusive than that used by Repenning & Tedford (1977) who included all non-odobenines in this subfamily. Deméré’s (1994b: 103) definition was based on “the most recent ancestor of *Pontolis* and *Dusignathus*,” a grouping that has been found to be paraphyletic by all later analyses (Kohno, 2006; Boessenecker & Churchill, 2013; Tanaka & Kohno, 2015; this study). Kohno (2006) proposed an amended phylogenetic definition for the Dusignathinae as “a clade containing the most recent common ancestor of *Dusignathus* and *Gomphotaria* and all of its descendants.” Since that time the concept of the Dusignathinae has been restricted to those two genera, but even the amended definition becomes problematic in light of recent analyses that show that it is paraphyletic and even question the monophyly of the genus *Dusignathus* (Boessenecker & Churchill, 2013; Tanaka & Kohno, 2015; this study). In light of this history, we do not propose a phylogenetic definition for Dusignathinae and question the further use of this name as the only way to ensure its monphyly is to restrict it to *D. santacruzensis.*

Setting aside “imagotariines” and “dusignathines,” there are three groupings (Odobenidae, Odobeninae, and Odobenini) that have crystallized in their usage over the past 14–50 years and are consistent with the phylogenetic definitions proposed by Deméré (1994b). We propose slightly amended definitions for two of these names by making them branch-based instead of node-based. We also propose a new name for an often-discussed and well-supported clade of odobenids. The phylogenetic names used in this study are listed below.

### Neodobenia, new clade name

**Definition**—The least inclusive clade that includes *Odobenus rosmarus* (Linnaeus, 1758), *Dusignathus seftoni* Deméré, 1994a, and *Gomphotaria pugnax* Barnes & Raschke, 1991.

**Figure 14:**
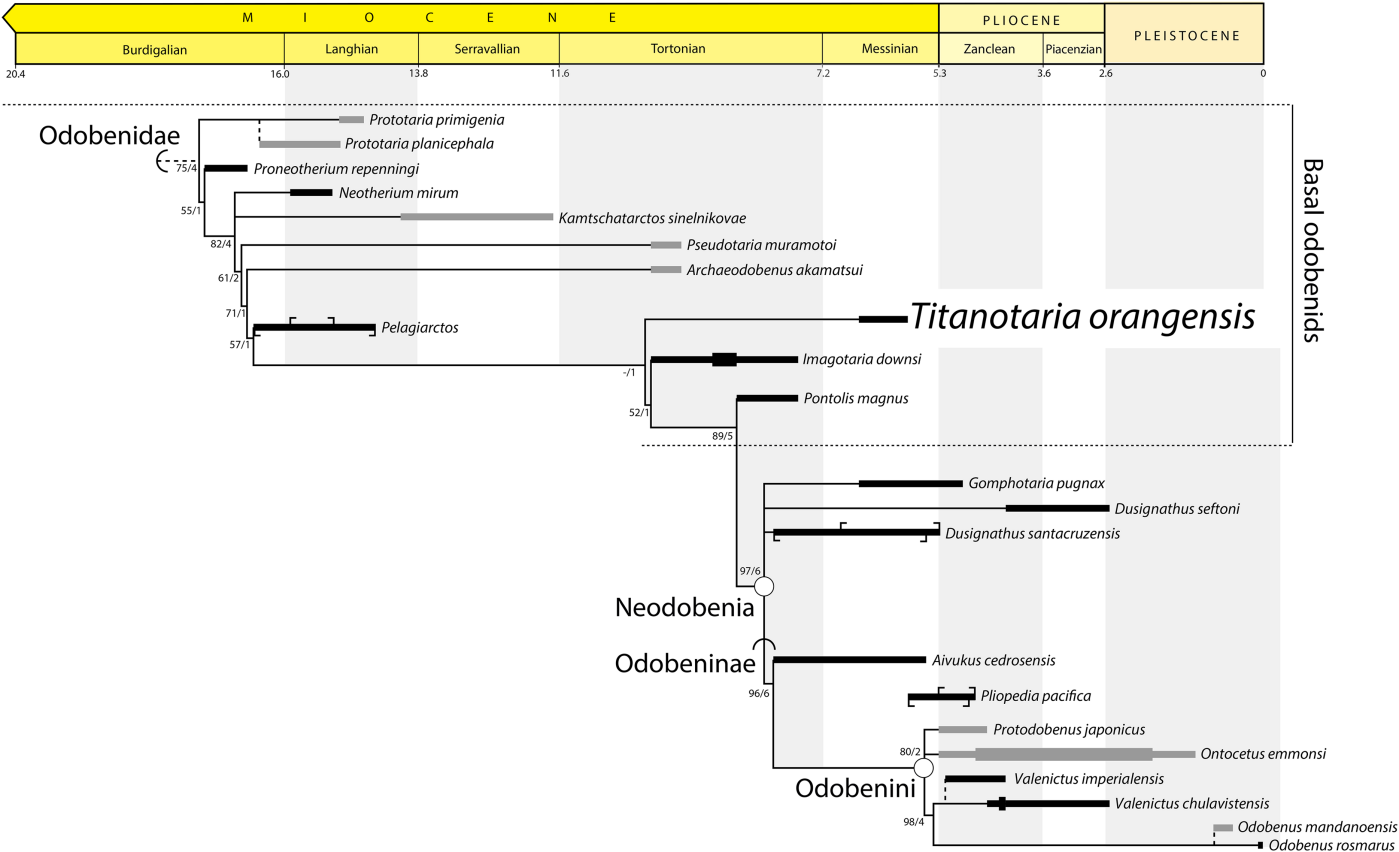
Preferred phylogenetic tree showing position of *Titanotaria orangensis* n. gen. et sp. (OCPC 11141). A stratigraphically-constrained strict consensus of 27 trees from the analysis with ordered characters. Outgroups (non-odobenids) not shown. Node- (circle) and branch-based (semicircle) phylogenetic names and bootstrap/decay values indicated. The length of ranges indicates age uncertainty for the formation, not taxon duration, with tick marks indicating the limits of specific units. In cases where formation ages do not overlap we show a known range (thick line). Taxa that do not occur in East Pacific formations are dark grey. Non-analyzed congeners indicated by dashed lines, with *Pliopedia pacifica* placed near the Odobenini. The lineage of *Valenictus* is extended to accommodate a specimen from the Purisima Formation. *Protodobenus japonicus* is sister to Odobenini in some trees.

**Reference phylogeny**—Figure 14 of this study.

**Etymology**—*Neo-* for “new;” -*odobenia* for Odobenidae.

**Comments**—One of the best supported nodes of our analysis unites the clade Odobeninae (see below) with the “dusignathines” (in the recent usage sense of a grade or clade that includes *G. pugnax* and *Dusignathus* spp., but not *P. magnus*). We do not use *D. santacruzensis* to define Neodobenia because it is poorly known, and in any case is always either sister to *D. seftoni* or else more closely related to *O. rosmarus* than *D. seftoni* in all analyses. Because this clade unites two important groupings of odobenids, and is evidently worth talking about (see mentions in Deméré, 1994b; Kohno, 2006; Boessenecker & Churchill, 2013), we feel it is useful to name. Neodobenia is diagnosed by single-rooted, simplified teeth. Most neodobenians are thought to have tusks except for *P. japonicus* and possibly *A. cedrosensis* (broken canines). We refer to all taxa on the stem of Neodobenia, informally, as “basal odobenids.”

### Odobenidae Allen, 1880, converted clade name

**Definition**—All taxa more closely related to *Odobenus rosmarus* (Linnaeus, 1758) than to *Phoca vitulina* Linnaeus, 1758 or *Otaria byronia* (De Blainville, 1820).

**Reference phylogeny**—Figure 14 of this study.

**Comments**—The first phylogenetic definition for Odobenidae was provided by Deméré (1994b: 102): “The Odobenidae are defined as the monophyletic group containing the most recent common ancestor of *Neotherium* and *Odobenus* and all of its descendants.” Later, Berta, Sumich & Kovacs (2006: 496) provided an amended definition for the Odobenidae: “Monophyletic group containing the common ancestor of stem odobenids *Imagotaria*, *Kamtschatarctos*, *Neotherium*, *Proneotherium*, *Pseudotaria*, *Prototaria*, *Pelagiarctos*, and crown odobenids and all of their descendants including *Aivukus*, *Dusignathus*, *Gomphotaria*, *Ontocetus*, *Pliopedia*, *Pontolis*, *Protodobenus*, and *Valenictus* and *Odobenus*.” This definition is problematic because it misapplies the crown clade concept (meant to be defined by most recent common ancestor of extant species) to apply to a clade name that is defined by extinct taxa. Both the Deméré (1994b) and Berta, Sumich & Kovacs (2006) definitions have the same content as a total clade name (Cantino & De Queiroz, 2014) for the extant *Odobenus rosmarus*. Therefore, we propose a branch-based Odobenidae, which is consistent with recent usage and complements the construction of its sister taxon, the branch-based Pan-Otariidae Gill, 1866 (Velez-Juarbe, 2017).

### Odobeninae Mitchell, 1968, converted clade name

**Definition**—All taxa more closely related to *Odobenus rosmarus* (Linnaeus, 1758) than to *Dusignathus santacruzensis* Kellogg, 1927, *Dusignathus seftoni* Deméré, 1994a, or *Gomphotaria pugnax* Barnes & Raschke, 1991.

**Reference phylogeny**—Figure 14 of this study.

**Comments**—The first phylogenetic definition for Odobeninae was provided by Deméré (1994b: 103): “The Odobeninae are here defined as the monophyletic group containing the most recent common ancestor of *Aivukus* and *Odobenus* and all of its descendants.” This definition includes all those species more closely related to *O. rosmarus* than to “dusignathines” and so we propose a branch-based Odobeninae.

### Odobenini Deméré, 1994b

**Definition**—”The monophyletic group containing the most recent common ancestor of *Alachtherium* [*Ontocetus emmonsi*] and *Odobenus*” (Deméré, 1994b: 104). The type species of *Alachtherium* is *Alachtherium cretsii* Du Bus, 1867, which was later referred to *Ontocetus emmonsi* Leidy, 1859, by Kohno & Ray (2008).

**Reference phylogeny**—Deméré (1994b: fig. 1).

**Comments**—Deméré (1994b) included all odobenines except *Aivukus cedrosensis* Repenning & Tedford, 1977 in his node-based definition of Odobenini. We do not propose a branch-based or otherwise emended phylogenetic definition for this name because it is less clear that this would capture the original intent (at the time it was coined, Odobenini was diagnosed by 14 unequivocal synapomorphies) and usage. Shortly after Deméré (1994b) was published, *Protodobenus japonicus* Horikawa, 1995, was described from the early Pliocene of Japan (published in parallel, neither paper cited each other). *Protodobenus japonicus* has been placed in the Odobenini by some later authors (Deméré, Berta & Adam, 2003) and not others (Boessenecker & Churchill, 2013). Our phylogenetic analysis alternatively resolves this taxon as the sister to *O. emmonsi* within Odobenini or as the sister taxon to Odobenini.

### Richness curve results

**Figure 15:**
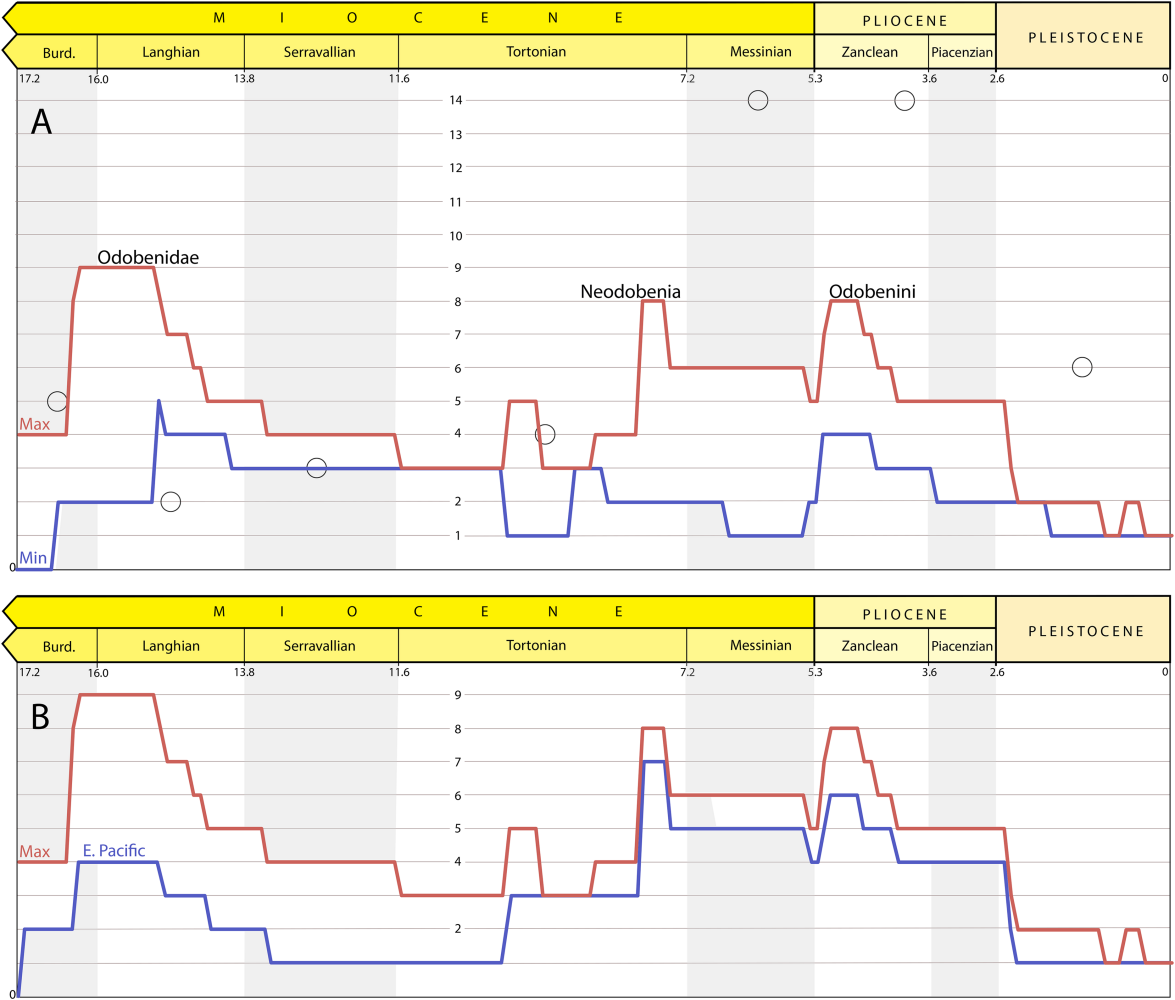
Richness estimates for Odobenidae. (A) Maximum (red) and minimum (blue) lineage counts accounting for ghost lineages and stratigraphic uncertainty. Peaks on the maximum richness curve labelled with corresponding clade originations. Open circles are from stage-binned analysis that includes undescribed specimens (includes both “small- and large-bodied” forms; Boessenecker & Churchill, 2018). (B) Maximum overall (red) and maximum of trees with East Pacific taxa only (blue) lineage counts.

The results of the maximum and minimum lineage count are shown in Fig. 15 (top). The maximum lineage count shows peaks near the base of Odobenidae, Neodobenia, and Odobenini. The lineage count is as high as nine in the Langhian and eight in the Messinian and as low as three and four in the intervening stages (Serravallian and Tortonian); the period of low lineage counts is between ∼15 and 8 Ma. After the Miocene, the maximum linage count decreases with a precipitous drop at the end of the Pliocene.

The minimum lineage count reaches a high of four briefly in the Langhian and throughout the Zanclean. The difference between the maximum and minimum curves is almost entirely determined by stratigraphic uncertainty because maximum lineage counts among the 27 trees were identical for 107 of the 173 time slices (61%) and within one for the other 67 (39%).

The results of the maximum lineages count when all taxa from outside the East Pacific were pruned from the trees is shown in Fig. 15 (bottom). When compared to the maximum global curve, the difference between the two curves is the relative input of lineages from outside the East Pacific. After 10 Ma, the richness curve is dominated by East Pacific lineages as shown by the close tracking of the two curves.

## Discussion

Our assessment of odobenid richness takes into account stratigraphic uncertainty and ghost lineages at 0.1 Ma intervals, giving insights into the timing of diversification events from a phylogenetic perspective. The overall pattern from our maximum richness curve (Fig. 15) generally conforms to the pattern shown by the stage-binned analysis of Boessenecker & Churchill (2018) with a few exceptions. Because that previous analysis incorporated undescribed material and used larger bins, we only highlight instances where our analysis estimates greater richness or the possibility of a different timescale for richness changes.

Our maximum diversity curve shows a peak near the origin of Odobenidae ∼16–15 Ma (∼early Langhian) that is higher than that of Boessenecker & Churchill (2018). In our analysis, this peak is determined, in part, by the fact that this is a time when multiple taxa from both the East and West Pacific are known. Another factor is the antiquity and phylogenetic position of *Pelagiarctos* sp., which necessitates ghost lineages for more basal, but younger, taxa (Fig. 14). *P. thomasi* is known from a single jaw (Sharktooth Hill Bonebed, Round Mountain Silt), and so was excluded from the first two cladistic analyses of odobenids (Deméré, 1994b; Kohno, 2006). Boessenecker & Churchill (2013) describe and refer additional jaw material from the “Topanga” Formation to *Pelagiarctos* sp. and include it in an updated matrix. They found it to be a sister taxon to *I. downsi* based on character states that at the time were unequivocal synapomorphies, but were later found in *T. orangensis* (c. 59 [1], 67[1]) and *A. akamatsui* (c. 67[1]). A *Pelagiarctos*-based OTU was excluded from the latest phylogenetic analysis of odobenids (Tanaka & Kohno, 2015). Those authors include a section on the “Enigmatic odobenid *Pelagiarctos*,” where they argue that *Pelagiarctos* is still too poorly known to be included in phylogenetic analyses. The lack of additional material referable to *Pelagiarctos* is vexing considering it is known from two formations (including at least one bonebed). A recently described specimen from the Sharktooth Hill Bonebed (Velez-Juarbe, in press) suggests that, despite the antiquity of the site (15.9-15.2 Ma) it may include more crownward odobenids. A partial jaw (LACM 135920) was hypothesized to be a new taxon more closely related to *P. magnus* than to other basal odobenids, i.e., even more crownward than *Pelagiarctos*. Unfortunately, the incomplete nature of LACM 135920 precludes inclusion in a cladistic analysis. It remains to be seen whether the phylogenetic position of *Pelagiarctos* and LACM 135920 should be refined or if, as requisite ghost lineages suggest, early Langhian odobenids were even more diverse than currently thought.

After about 15 Ma, the fossil record of odobenids becomes much more incomplete. For example, there is just one described taxon from the Serravallian (13.8–11.6 Ma), *K. sinelkovae*, which is poorly constrained stratigraphically (Appendix 1) and in need of redescription (Boessenecker & Churchill, 2013: 12). The recently described *N. arandai* (Velez-Juarbe & Salinas-Márquez, 2018) may also be Serravallian, but its age is poorly constrained (15.7–9.2 Ma). The low number of odobenids is in contrast to the non-odobenid pinniped *Allodesmus*, which may have three to four described taxa in the Serravallian (Boessenecker & Churchill, 2018; Tonomori et al., 2018). Boessenecker & Churchill (2018) suggest the possibility of competitive displacement of odobenids and *Allodesmus*, which seems plausible given their undeniable similarity in size, morphology, and presumably aspects of their ecology. Debey & Pyenson (2013) suggest that *Allodesmus* is a deep-diving taxon, which may also be a factor that may favor their preservation in certain formations over odobenids. The number of observed odobenid lineages remains low until the late Tortonian or early Messinian (8–7 Ma), coincident with both the extinction of *Allodesmus* (Boessenecker & Churchill, 2018) and the diversification of neodobenians.

The exact timing of the neodobenian diversification event is unclear, the potentially oldest members (*A. cedrosensis* and *D. santacruzensis* from the Almejas Formation) have poor temporal constraints (8.0–5.5 Ma, Appendix 1). As such, our maximum richness curve shows a potential peak before the Messinian (7.2–5.3 Ma), earlier than where Boessenecker & Churchill (2018) show their major increase in richness. An undescribed specimen referred to *Gomphotaria* from the Towsley Formation was reported from the Tortonian by Boessenecker & Churchill (2018) would seemingly confirm a pre-Messinian origin for neodobenians. However, parts of the Towsley Formation are considered to be latest Miocene or even Pliocene in age by other authors (Squires, 1991; Yeats, Huftile & Stitt, 1994; 6.5–5.0 Ma), which means there are no definitive pre-Messinian neodobenians. Therefore, with the available data, we can recognize a provisional timescale for the transition from odobenid assemblages that include just basal odobenids from Empire and older formations (>7 Ma), to a mixture of basal odobenids and neodobenians from the Capistrano and basal Purisima Formations (∼7–5 Ma), and then just neodobenians from all younger units (<5 Ma). In this regard, *T. orangensis* (6.6–5.8 Ma) is an important addition to our understanding of odobenid assemblages through time because it is one of the best-known and latest-surviving basal odobenids.

The Messinian peak of odobenid richness coincides with the evolution of more specialized feeding systems of neodobenians, including the evolution of tusks and molluscivory, which displaced them from basal odobenids ecologically. Basal odobenids go extinct before the Pliocene, and odobenid richness by that time is dominated by molluscivorous odobenines (Odobenini). Odobenids show a decrease in known richness in the Piacenzian by and ultimately a precipitous drop in the Pleistocene along with other taxa. Recent studies have attributed this extinction event to the reduction of the neritic zone, for example, shallow marine foraging areas, utilized by the molluscivorous Odobenini (Boessenecker, 2013a, 2017; Pimiento et al., 2017).

In contrast to the maximum lineage count for odobenids, the minimum lineage counts remain more stable (Fig. 15). Small peaks near the base of clade origins (Odobenidae, Neodobenia, Odobenini) mirror that of the maximum lineage richness curve. Aside from another peak in the Zanclean, which coincides with the diversification of Odobenini, this analysis alone cannot refute that the standing richness of odobenids was more than three species for most of their history (but see below). Two factors can account for this low estimate relative to the stage-binned analysis of Boessenecker & Churchill (2018). First, we have excluded abundant undescribed material (Appendix 2). Second, since each bin was calculated independently, it means that a lineage with an uncertain range can be considered to not have diverged at one 0.1 Ma interval and then be considered extinct in the next, whichever resulted in the lowest possible lineage count. In this way our curve shows the minimum at any given time, but no single time-constrained tree matches the entire minimum curve. With this in mind, a key comparison for our minimum estimate of odobenid richness is formations that include more than one species of odobenid. Most formations have just one or two species known, but starting in the late Tortonian we begin to see formations with up to four species, although some of these have not been formally described (Appendix 2). For example, with the addition of *T. orangensis* and unpublished specimens (Barboza et al., 2017; Velez-Juarbe, 2017; Appendix 2), the Oso Member might have four species of odobenids. The same is potentially true for the contemporaneous basal Purisima Formation and slightly older Empire Formation (Boessenecker, 2017; Appendix 2). Although these counts are based on undescribed material, they complement the increased richness suggested by the maximum curve of our conservative analysis and previous studies.

The largest discrepancy between the maximum and minimum estimates is during the Messinian diversification of neodobenians (nine vs. one lineage) and can be attributed to aforementioned stratigraphic uncertainty surround the origination of Neodobenia. For example, if *P. magnus* and *T. orangensis* went extinct before the deposition of the Almejas Formation, then the intervening time interval would just include a single estimated lineage, the ghost lineage of Neodobenia. This example demonstrates the large impact of stratigraphic uncertainty as a bias to increase and potentially overestimate lineage counts.

The most important bias to consider when interpreting odobenid richness is the temporal and geographic distribution of odobenid-bearing deposits. Our analysis of maximum lineage counts from the East Pacific closely matches that of the corresponding global curve, indicating that after 9.0 Ma the overall shape of the curve is largely driven by East Pacific taxa. Therefore, the pattern of increased odobenid richness seen here could be influenced by regional depositional patterns in the East Pacific. Kloess & Parham (2017) show that in California, where most of the formations in this study are from, the fossil record between 15 and 10 Ma is characterized by just a few formations that were deposited at upper to mid bathyal depths, whereas after this time many more deposits from shallow water are known. The deeper water deposits correspond to a time of low odobenid richness, which may reflect a reduced preservation potential. It remains to be tested how much the timescale for the transition from *Allodesmus*, to basal odobenids, to neodobenian assemblages reflects the timing of these depositional changes among regions.

A corollary of the large impact of East Pacific taxa on late Miocene richness estimates is that the West Pacific record is relatively poor (Fig. 1); there are no definitive records of West Pacific odobenids recorded between 9.5 and 5.3 Ma, including the time of highest estimated richness for odobenids in the East Pacific. The faunal list of Miyazaki et al. (1995) suggests that this record may be improved, but even then the record is sparse when compared with the amount of undescribed material from the East Pacific (Appendix 2). Given this unevenness, it is not possible to know whether the diversification events and assemblage transitions seen in the East Pacific happened in a similar way in the West Pacific. In light of the unevenness of the odobenid fossil record and stratigraphic uncertainty, estimates of odobenid richness and their correlations should be treated with caution. The amount of undescribed material mentioned in the literature (Appendix 2) shows great potential for future study. In addition to describing these specimens and placing them in a phylogenetic context, new discoveries and additional chronostratigraphic work on odobenid-bearing sites will help test, refine, and refute the patterns presented here.

## Conclusions

We describe *Titanotaria orangensis* (gen. et. sp. nov.), a new species of walrus (odobenid) from the upper Miocene Oso Member of the Capistrano Formation of Orange County, California. This species is important because: (1) It is one of the best-known and latest-surviving basal odobenids; (2) It raises the number of reported odobenid taxa from the Oso Member to four species making it one of the most rich walrus assemblages known (along with the basal Purisima of Northern California); (3) It is just the second record of a tuskless walrus from the same unit as a tusked taxon. Our phylogenetic analysis places *T. orangensis* as sister to a clade that includes *I. downsi*, *P. magnus*, *Dusignathus* spp., *G. pugnax*, and Odobeninae. We propose new branch-based phylogenetic definitions for Odobenidae, Odobeninae, and a new node-based name (Neodobenia) for the clade that includes *Dusignathus* spp., *G. pugnax*, and Odobeninae. A richness analysis at the 0.1 Ma level that incorporates stratigraphic uncertainty and ghost lineages demonstrates maximum peaks of richness (up to eight or nine coeval lineages) near the base of Odobenidae, Neodobenia, and Odobenini. A more conservative minimum curve demonstrates that standing richness may have been much lower than the maximum richness estimates that are biased by stratigraphic uncertainty. Overall the odobenid fossil record is uneven, with large time slices of the record missing on either side of the Pacific Ocean at some times and biases from the preserved depositional environments at other times. We recognize a provisional timescale for the transition of an East Pacific odobenid assemblages that includes “basal odobenids” (stem neodobenians) from the Empire and older formations (>7 Ma), to a mixture of basal odobenids and neodobenians from the Capistrano and basal Purisima (7–5 Ma), and then just neodobenians from all younger units (<5 Ma). The large amount of undescribed material will add new taxa and range extensions for existing taxa, which will likely change some of the patterns we describe.

## Appendix 1. List of Valid Taxa with Chronostratigraphic Assessments

***Aivukus cedrosensis*Repenning & Tedford, 1977**

**Age range** 8.0–5.5 Ma.

**Occurrence:** East Pacific, lower part of the Almejas Formation, Cedros Island, Mexico.

**Maximum age:** Boessenecker & Churchill (2015, suppl. info.) report a maximum age of the Almejas Formation of ∼8 Ma based on Barnes (1998). Barnes (1998) tentatively reports the maximum age as ∼9 Ma and notes correlations with other upper Miocene formations. Barnes (2008) later refines the maximum age to ∼8 Ma, which we use here but note that more detailed faunal correlations can provide a more refined age.

**Minimum age:** Boessenecker & Churchill (2015, suppl. info.) estimate the minimum age of the Almejas Formation to be 5.7 Ma based on isotopic analyses of volcanic beds above the unit (Sawlan & Smith, 1984). Because the age of the volcanic bed is 5.7 ± 0.2 Ma, we report the minimum possible age 5.5 Ma.

***Archaeodobenus akamatsui*Tanaka & Kohno, 2015**

**Age range:** 10.0–9.5 Ma.

**Occurrence:** West Pacific, Ichibangawa Formation, Hokkaido, Japan.

**Maximum age:** Fission track dating derived from a tuff layer within the underlying Subestsu Formation are dated at approximately 13 ± 1 Ma (Tsuji et al., 1992). However, we follow Tanaka & Kohno (2015) who accept a maximum age of 10.0 Ma based on the stratigraphic study of Takano et al. (1996).

**Minimum age:** We follow Tanaka & Kohno (2015) who accept a minimum age of 9.5 Ma based on the stratigraphic study of Takano et al. (1996).

***Dusignathus santacruzensis*Kellogg, 1927**

**Age range:** 8.0–5.3 Ma.

**Occurrence:** East Pacific, Purisima Formation, California, USA and lower part of the Almejas Formation, Cedros Island, Mexico.

**Maximum age:** As for *A. cedrosensis* (see above).

**Minimum age:** The minimum age is based on the basal Purisima Formation (6.9–5.3 following Boessenecker, 2017).

***Dusignathus seftoni*Deméré, 1994a**

**Age range:** 3.6–2.5 Ma.

**Occurrence:** East Pacific, “lower member” (*sensu* Deméré, 1983), San Diego Formation, California, USA.

**Maximum age:** The maximum age is 4.2 Ma based on Vendrasco et al. (2012).

**Minimum Age:** The minimum age is based on the occurrence of the planktonic foraminifers that indicate deposition during the California margin planktonic foraminiferal zone 6 (Kennett, Rozo-Vera & Machain Castillo, 2000) which has an age range from 3.25 to 2.5 Ma (Kucera & Kennett, 2000).

***Gomphotaria pugnax*Barnes & Raschke, 1991**

**Age range:** 6.6–4.9 Ma.

**Occurrence:** East Pacific, unnamed siltstone member, Capistrano Formation, California, USA.

**Maximum age:** The maximum age is based on the maximum age of the Oso Member, 6.6 Ma (Barboza et al., 2017), which grades laterally into the basal part of the unnamed siltstone member.

**Minimum age:** The minimum age is based on diatom biostratigraphy from the vertebrate bearing layers (Deméré & Berta, 2005).

***Imagotaria downsi*Mitchell, 1968**

**Age range:** 10.0–7.6 Ma.

**Occurrence:** East Pacific, Monterey Formation localities include the Great Lakes Carbon Co. Mine and the Celite Company No. 38 Quarry near Lompoc, California, USA. *Imagotaria downsi* is also known from the upper sandstones of the Santa Margarita Formation of California. Other specimens of *I. downsi* are reported from the Towsley and Monterey Formations of California, however, this material is largely fragmentary so they are not considered when constraining the age range of this taxon following Boessenecker & Churchill (2015, suppl. info.).

**Maximum age:** Our maximum age determination for *I. downsi* follows that of Boessenecker & Churchill (2015). The maximum age is based on the age of the upper sandstones of the Santa Margarita Formation. The Santa Margarita Formation is summarized by Repenning & Tedford (1977) to have an age range from 12 to 9 Ma based on fossil invertebrates and equid teeth. Referred specimens of *I. downsi* were recovered from the lower limit of the upper sandstones. Repenning & Tedford (1977) correlated that particular layer to those found above and below the Moraga Formation in the Berkeley Hills of California based on the presence of *Hipparion* De Christol, 1832. This Moraga Formation is dated at 10 Ma (Repenning & Tedford, 1977).

**Minimum age:** The minimum age is based on biostratigraphic data from the Monterey Formation, Celite Company No. 38 Quarry near Lompoc. Diatoms yielded from this locality are indicative of Schrader’s Diatom Zone XI (Repenning & Tedford, 1977). The limits of Schrader’s Diatom Zone XI is equivalent to the *Thalassiosira antiqua* zone (Barron, 1986), which is based on the FO of *T. antiqua* and the LCO of *Thalassionema schraderi* (8.6–7.6 Ma fide Gradstein et al., 2004).

**Minimum range:** Because *I. downsi* is known from multiple formations with non-overlapping ages, the minimum possible age range is from 9.0 to 8.6 Ma, with 9.0 Ma being the estimated age youngest age of the Santa Margarita Formation and 8.6 Ma being the maximum estimated age of the Monterey Formation at Celite Company No. 38 Quarry near Lompoc.

***Kamtschatarctos sinelnikovae*Dubrovo, 1981**

**Age range:** 14.1–11.6 Ma.

**Occurrence:** West Pacific, Etolon Formation, Kamchatka, Russia.

**Maximum age:** The maximum age is based on the underlying Kakert Formation, which is shown to include at least part of chron C5Acn (Shilo & Minyuk, 2006; Gladenkov, 2013), the base of which is 14.07 Ma (Hilgen, Lourens & Van Dam, 2012).

**Minimum age:** The minimum age is based on the minimum age of the middle Miocene, i.e., the top of the Serravallian (Hilgen, Lourens & Van Dam, 2012).

***Neotherium mirum*Kellogg, 1931**

**Age range:** 15.9–15.2 Ma.

**Occurrence:** East Pacific, Sharktooth Hill Bonebed, Round Mountain Silt Member, Temblor Formation, California, USA.

**Maximum and minimum age:** The maximum and minimum ages are based on Pyenson et al. (2009) who showed that the bonebed was deposited between 15.9 and 15.2 Ma using paleomagnetism (Prothero, Francisco & Denke, 2008) and biostratigraphy (Kohno et al., 1995).

***Odobenus mandanoensis* Tomida, 1989**

**Age range:** 0.8–0.5 Ma.

**Occurrence:** Western Pacific, Mandano Formation, Kazusa Group, Boso Peninsula, Japan.

**Maximum age:** The maximum age is based on the Byakubi-E tephra within the underlying Kokumoto Formation, which is dated at 0.773 Ma (Kazaoka et al., 2015).

**Minimum age:** The minimum age is based on the youngest limit of the Kazusa Group (Ito & Katsura, 1992; Kazaoka et al., 2015).

***Odobenus rosmarus* (Linnaeus, 1758)**

**Age range:** 0.1–0.0 Ma.

**Occurrence:** The modern walrus (*O. rosmarus*) is restricted to Arctic regions, but the fossil record extends as far south as the San Francisco Bay of California in the Pleistocene (Harington, 1984; Dyke et al., 1999).

**Maximum age:** The oldest fossil record of *Odobenus rosmarus* comes from a glaciomarine clay found in British Columbia, Canada dated to 70,000 years ago (Harington & Beard, 1992). Older fossils assigned to *Odobenus* from Japan and Russia are reviewed by Boessenecker & Churchill (2015, suppl. info.). The oldest fossil record of *Odobenus* is reported by Kohno et al. (1995) which is dated at 2.7–2.0 Ma based on associated diatoms of the *Neodenticula koizumii* zone. Given that the sister taxon, *Valenictus*, is thought to be as old as 4.2 Ma (minimum possible age of *V. imperialensis*), these fossils are effectively accommodated by counting ghost lineages.

**Minimum age:** It is extant.

***Ontocetus emmonsi*Leidy, 1859**

**Age range:** 5.3–1.1 Ma.

**Occurrence:** Fossil occurrences of *O. emmonsi* range from the eastern United States, Europe, and Morocco (Kohno & Ray, 2008; Vélez-Juarbe, Wood & Pimiento, 2016; Boessenecker, Boessenecker & Geisler, 2018). Fossils from Japan have been referred to the genus Ontocetus (Kohno, Narita & Koike, 1998).

**Maximum age:** The maximum age is based on oldest age of the Palmetto Fauna of the upper Bone Valley Formation (Morgan, 1994; Webb et al., 2008). The maximum age for this unit has been given as 5.8 Ma (Boessenecker, Boessenecker & Geisler, 2018) because that is the maximum age of the latest Hemphillian (Hh4, 5.8–4.9 Ma, Hilgen, Lourens & Van Dam, 2012), but Morgan (1994) demonstrates an absence of Messinian marine mammals in the Palmetto Fauna, and placed the lower limit at the base of the Zanclean (also the base of the Pliocene).

**Minimum age:** The minimum age is based on a recently described specimen from the Austin Sand Pit of South Carolina, dated to 1.8–1.1 Ma (Boessenecker, Boessenecker & Geisler, 2018).

**Minimum range:** The minimum range of *O. emmonsi* is from 4.9 to 1.8 Ma, which is the youngest age of the Upper Bone Valley Formation (Palmetto Fauna; 5.3–4.9 Ma; Morgan, 1994; Hilgen, Lourens & Van Dam, 2012) and the oldest age of the possible age from the Austin Sand Pit (1.8–1.1 Ma; Boessenecker, Boessenecker & Geisler, 2018).

***Pelagiarctos*Barnes, 1988**

**Age range:** 16.5–14.5 Ma.

**Occurrence:** East Pacific, Sharktooth Hill Bonebed, Round Mountain Silt Member, Temblor Formation, California, USA (Barnes, 1988) and “Topanga” Formation, California, USA (Boessenecker & Churchill, 2013).

**Maximum age:** The maximum age is based on benthic foraminifera found in the “Topanga” Formation (Raschke, 1984) that correspond with the early Relizian California stage, which begins at 16.5 Ma (Gradstein et al., 2004).

**Minimum age:** The minimum age is based on benthic foraminifera found in the “Topanga” Formation (Raschke, 1984) that correspond with the early Luisian California stage, which ends at 14.5 Ma (Gradstein et al., 2004).

**Comments:** Here, we provisionally combine *P. thomasi* with “*Pelagiarctos* sp.” of Boessenecker & Churchill (2013) since they can’t be definitely distinguished. *Pelagiarctos thomasi* is from the Sharktooth Hill Bonebed (15.8–15.2 Ma, see *N. mirum* above).

***Pliopedia pacifica* (Kellogg, 1921)**

**Age range:** 5.8–4.7 Ma.

**Occurrence:** East Pacific, Paso Robles and Etchegoin Formations, California, USA.

**Maximum age:** For the Etchegoin Formation, Loomis (1990) gives a basal age of 5.5 Ma based on diatoms, but Loomis (1992) bases the maximum age on a tuff from within a lower formation (∼7.0 Ma). The presence of cervid deer from the Etchegoin (Merriam, 1915) can help constrain its age since cervids are not known in North America before Hh4 (Tedford et al., 2004), the base of which is 5.8 Ma (Hilgen, Lourens & Van Dam, 2012). The base of the Paso Robles Formation is not well constrained, but is thought to be Pliocene (Repenning & Tedford, 1977), which gives a maximum age of 5.333 Ma (Cohen, Harper & Gibbard, 2017) for the Paso Robles Formation.

**Minimum Age:** The age of the *P. pacifica* from the Paso Robles Formation is usually given as 6–5 Ma (Deméré, 1994b; Boessenecker, 2013b), ultimately based on an ad hoc assessment by Repenning & Tedford (1977) which considered the level of morphological differentiation of *P. pacifica*. Later studies show that the Huichica Tuff (4.76 ± 0.02) occurs in the Paso Robles Formation, the type specimen was collected at the base of the Paso Robles so we can constrain its minimum possible age to 4.74 Ma. This is slightly younger than the youngest possible age of the specimens from the Etchegoin Formation. Repenning & Tedford (1977) report that the Etchegoin specimens were collected below a tuff that is later identified as the Lawlor Tuff by Loomis (1992). The Lawlor Tuff has a youngest possible age of 4.823 Ma (4.834 ± 0.011 Ma; Sarna-Wojcicki et al., 2011).

***Pontolis magnus* (True, 1905)**

**Age range:** 8.6–7.6 Ma.

**Occurrence:** East Pacific, Empire Formation, Oregon, USA.

**Maximum and minimum age:** The maximum and minimum ages are based on diatom biostratigraphy. The study of Barron (1986) showed that the Empire Formation is in the *Thalassiosira antiqua* zone, which is based on the FO of *T. antiqua* and the LCO of *Thalassionema schraderi* (8.6–7.6 Ma fide Gradstein et al., 2004). Prothero, Lau & Armentrout (2001) suggested that the Empire Formation was deposited between 8.7 and 6.5 Ma based on paleomagnetism and biostratigraphy, but did not include the addendum of Barron (1981, fig. 7) which restricted the type section to Schrader’s Diatom Zone XI. This zone is equivalent to the *Thalassiosira antiqua* zone (Barron, 1986), which is based on the FO of *T. antiqua* and the LCO of *Thalassionema schraderi* (8.6–7.6 Ma fide Gradstein et al., 2004).

***Proneotherium repenningi* Barnes in Kohno, Barnes & Hirota 1995**

**Age range:** 17.3–16.6 Ma.

**Occurrence:** East Pacific, “Iron Mountain Bed” of the Astoria Formation, Oregon, USA.

**Maximum and minimum age:** The maximum and minimum age is based on Prothero et al. (2001) who showed that the “Iron Mountain Bed” was deposited between 17.3 and 16.6 Ma based on paleomagnetism and biostratigraphy.

***Protodobenus japonicus*Horikawa, 1995**

**Age range:** 5.3–4.5 Ma.

**Occurrence:** West Pacific, lower member of the Tamugigawa Formation, Niigata, Japan.

**Maximum and minimum age:** The maximum and minimum ages are based on estimates from Takano (1990).

***Prototaria planicephala*Kohno, 1994**

**Age range:** 16.4–15.1 Ma.

**Occurrence:** West Pacific, Moniwa Formation, Miyagi, Japan.

**Maximum and minimum age:** The maximum and minimum ages are based on planktonic foraminiferal zone N8 (Kohno, 1994; Gradstein et al., 2012).

***Prototaria primigena*Takeyama & Ozawa, 1984**

**Age range:** 15.1–14.7 Ma.

**Occurrence:** West Pacific, muddy fine-grained sandstone unit of the Shimo Formation, Fukui, Japan.

**Maximum and minimum age:** The maximum and minimum ages are based on planktonic foraminifera that correspond to the lower part of Zone N9 Kohno et al. (1995), which ranges from 15.10 to 14.66 Ma (Gradstein et al., 2012).

***Pseudotaria muramotoi*Kohno, 2006**

**Age range:** 10.0–9.5 Ma.

**Occurrence:** West Pacific, Ichibangawa Formation, Hokkaido, Japan.

**Maximum age:** As for *A. akamatsui*.

**Minimum age:** As for *A. akamatsui*.

***Titanotaria orangensis* gen. et. sp. nov.**

**Age range:** 6.6–5.8 Ma.

**Occurrence:** East Pacific, Oso Member, Capistrano Formation, California, USA.

**Maximum and minimum age:** The maximum and minimum ages are based on Barboza et al. (2017).

***Valenictus chulavistensis*Deméré, 1994a**

**Age range:** 4.5–2.5 Ma.

**Occurrence:** East Pacific, “lower member” (sensu Deméré, 1983) of the San Diego Formation and San Joaquin Formation of California, USA.

**Maximum age:** The maximum age (4.5 Ma) is based on the San Joaquin specimen, which is about 4.5–4.3 Ma, constrained by the basal age of the San Joaquin Formation (Hosford Scheirer & Magoon, 2007) and a tuff bed in the San Joaquin Formation that was dated to 4.3 Ma (Repenning & Tedford, 1977).

**Minimum age:** The minimum age (2.5 Ma) is based on the San Diego Formation. The minimum age of that formation is determined by the occurrence of the planktonic foraminifers assigned to California margin planktonic foraminiferal zone 6 (Kennett, Rozo-Vera & Machain Castillo, 2000) which has an age range from 3.25 to 2.5 Ma (Kucera & Kennett, 2000). The maximum age of the San Diego Formation is 4.2 Ma (Vendrasco et al., 2012).

**Note:** We assume that *V. imperialensis* is a valid sister taxon to *V. chulavistensis* thereby necessitating a ghost lineage between the two species. We further extend the maximum age of the *Valenictus* lineage to 5.3 Ma following the report of a specimen from the Purisima Formation Boessenecker (2017) estimated to be between 5.3 and 4.9 Ma.

***Valenictus imperialensis*Mitchell, 1961**

**Age range:** 5.2–4.2 Ma.

**Occurrence:** East Pacific, the Deguynos Formation, California, USA.

**Maximum and minimum age:** The maximum and minimum ages are based on its occurrence in the Deguynos Formation, which is well dated by Dorsey et al. (2011).

## Appendix 2. List of Undescribed or Insufficiently Characterized Specimens that Have Been Mentioned in Recent Papers Since 2017, but Are Not Included in Our Richness Estimate. By Its Focus on Recent Papers Looking at Pinniped Diversity, This Is Not Meant to Be an Exhaustive List of Unpublished Odobenid Material. Taxa Are Listed Alphabetical Order By Formation (where Known)

### Capistrano Formation, California, USA

In addition to *G. pugnax* and *T. orangensis*, at least two undescribed taxa of odobenids have been mentioned from the Capistrano Formation (Barboza et al., 2017; Velez-Juarbe, 2017; Boessenecker & Churchill, 2018). Barboza et al. (2017) and Velez-Juarbe (2017) both list LACM 118967, and the latter lists LACM 150922, as representative specimens of these undescribed taxa. Boessenecker & Churchill (2018: suppl. data) indicate that Deméré, Berta & Adam (2003) list *P. magnus* from the Capistrano Formation, but those data are not given in that paper.

### Deguynos Formation, California, USA

Boessenecker & Churchill (2018: suppl. data) cite an abstract (Atterholt, Jefferson & Schachner, 2007) for the presence of a potential new species of *Valenictus* in the Deguynos Formation, the type strata of *V. imperialensis*.

### Empire Formation, Oregon, USA

Besides *P. magnus*, the Empire Formation is known to have additional taxa, although they are currently undescribed. Boessenecker & Churchill (2018: suppl. data) cite Deméré (1994b), who lists USNM 335594 and 335599 as “*Imagotaria* sp. cf. *I. downsi*,” an abstract (Sha[h]bazi et al., 2016), and personal observations to account for two undescribed taxa.

### Monterey Formation, California, USA

In addition to *I. downsi*, Velez-Juarbe (2017) lists two taxa from the Monterey Formation and provides two specimen numbers (LACM 123282 and 122444) as their representatives. Velez-Juarbe (2017) also mentions two specimens (LACM 4324, 17588) from the Valmonte Diatomite Member that he hypothesizes could be a new taxon. LACM 4324 was described by Lyon (1941) and referred to *P. magnus*.

### Purisima Formation, California, USA

Boessenecker, Perry & Schmitt (2014) and Boessenecker (2017) list four species of odobenids from the basal Purisima Formation. Currently *Dusignathus* is the only one that is described (Kellogg, 1927; Boessenecker, 2013b, 2017).

### San Diego Formation, California, USA

In addition to *D. seftoni* and *V. chulavistensis*, Boessenecker & Churchill (2018: suppl. data) cite Boessenecker (2013b) which provides a specimen (LACM 28432) and a preliminary diagnosis for a new taxon.

### Santa Margarita Sandstone, California, USA

In addition to *I. downsi*, Boessenecker & Churchill (2018: suppl. data) list two taxa “*Imagotaria* n. sp. 1 and 2.” One of these new species is based on “Desmatophocine A” of Barnes (1972), UCMP 85197. This taxon is incorrectly listed as coming from the Round Mountain Silt by Boessenecker & Churchill (2018: table 9). The other undescribed taxon is based on material described by Repenning & Tedford (1977) and is most likely USNM 23858.

### “Topanga” Formation, California, USA

In addition to *Pelagiarctos* sp., Velez-Juarbe (2017) mentions “*Neotherium* sp.” from the “Topanga” Formation of Orange County. The specimen number for this material is LACM 123713. Boessenecker & Churchill (2018: suppl. data) lists cf. *Imagotaria* citing personal observations.

### Towsley Formation, California, USA

Boessenecker & Churchill (2018: suppl. data) cite an abstract (Berkoff & Barnes, 1988) that diagnoses a new species of *Gomphotaria*. Although Berkoff & Barnes (1988) report the age of this specimen as being 10–9 Ma, which was later expanded upon by Whitmore & Barnes (2008), parts of the Towsley are considered Late Miocene or even Pliocene by other authors (Squires, 1991; Yeats, Huftile & Stitt, 1994; 6.5–5.0 Ma).

### Other Japanese Formations

Miyazaki et al. (1995, fig. 2) lists two odobenids in the late Messinian: “*Imagotaria* cf. *downsi*” and “Imagotariinae,” but only cites a reference for one of them (Imagotariinae, Nakagawa, 1994) which is missing from the literature cited section of that manuscript. We were given the full citation for the Nakagawa (1994) paper, as well as another (Aizu Fossil Research Group, 1985), during review, but were unable to evaluate them before resubmission.

## Supplemental Information

10.7717/peerj.5708/supp-1 Supplemental Information 1 Data matrix used for phylogenetic analysis. 25 taxon, 91 character, data matrix of based on Boessenecker and Churchill (2013) with the addition of *Titanotaria orangensis* and *Archaeodobenus akamatsui* and character 91 from Tanaka & Kohno (2015). Coding changes noted in Phylogenetic Methods.

## Institutional Abbreviations

LACM: Department of Vertebrate Paleontology, Natural History Museum of Los Angeles County, Los Angeles, California, USA
LACM Loc.: Vertebrate Paleontology Locality, Natural History Museum of Los Angeles County, Los Angeles, California, USA
LC: Ralph B. Clark (formerly “Los Coyotes”) Regional Park, Interpretive Center, Buena Park, California, USA
NMNS: National Museum of Nature and Science, Tokyo, Japan
OCPC: Orange County Paleontology Collection, John D. Cooper Center, Santa Ana, California, USA
SDNHM: San Diego Natural History Museum, San Diego, California, USA
SFMV: Shiga Fossil Museum, Vertebrate Collection, Matsumoto, Nagano, Japan
UCMP: University of California Museum of Paleontology, Berkeley, California, USA
USNM: Departments of Paleobiology and Vertebrate Zoology (Division of Mammals), National Museum of Natural History, Smithsonian Institution, Washington DC, USA.

## Additional Information and Declarations

### Data Availability

The following information was supplied regarding data availability: The data matrix used for phylogenetic analysis is provided as a Supplemental File.

### New Species Registration

The following information was supplied regarding the registration of a newly described species: Publication LSID: urn:lsid:zoobank.org:pub:7826C97C-3994-4519-89F0-603E6135F3DB. *Titanotaria* Magallanes, Parham, Santos & Velez-Juarbe LSID: urn:lsid:zoobank.org:act:7BED71E3-BB44-4AAD-8BD7-A7B6319C5565. *Titanotaria orangensis* Magallanes, Parham, Santos & Velez-Juarbe LSID: urn:lsid:zoobank.org:act:ADE1E563-CDB6-453E-B686-B0BC2345DFE4.

## References

[ref-1] Aizu Fossil Research Group (1985). On two fossil humeri of Otarioidea from the Shiotsubo Formation (Late Miocene) of Fukushima Prefecture, northeast Japan. Chikyu-Kagaku (Earth Science).

[ref-2] Allen JA (1880). History of North American Pinnipeds, a monograph of the walruses, sea-lions, sea-bears and seals of North America. U.S. Geological and Geographical Survey of the Territories Miscellaneous Publications.

[ref-3] Atterholt J, Jefferson G, Schachner E (2007). New marine vertebrates from the Yuha Member of the Deguynos Formation of Anza-Borrego Desert State Park. Journal of Vertebrate Paleontology.

[ref-4] Barboza MM, Parham JF, Santos G-P, Kussman BN, Velez-Juarbe J (2017). The age of the Oso Member, Capistrano Formation, and a review of fossil crocodylians from California. PaleoBios.

[ref-5] Barnes LG (1972). Miocene Desmatophocinae (Mammalia: Carnivora) from California. University of California Publications in Geological Sciences.

[ref-6] Barnes LG (1988). A new fossil pinniped (Mammalia: Otariidae) from the middle Miocene Sharktooth Hill Bonebed, California. Contributions in Science, Natural History Museum of Los Angeles County.

[ref-7] Barnes LG, Carranza-Castañeda O, Córdoba-Méndez DA (1998). The sequence of fossil marine mammal assemblages in México. Avances en Investigación. Paleontología de Vertebrados.

[ref-8] Barnes LG (2008). Miocene and Pliocene Albireonidae (Cetacea, Odontoceti), rare and unusual fossil dolphins from the eastern North Pacific Ocean. Natural History Museum of Los Angeles County Science Series.

[ref-9] Barnes LG, Raschke RE (1991). *Gomphotaria pugnax*, a new genus and species of late Miocene dusignathine otariid pinniped (Mammalia, Carnivora) from California. Contributions in Science, Natural History Museum of Los Angeles County.

[ref-10] Barron JA (1981). Marine diatom biostratigraphy of the Montesano Formation near Aberdeen, Washington. Pacific Northwest Cenozoic Biostratigraphy.

[ref-11] Barron JA, Casey RE, Barron JA (1986). Updated diatom biostratigraphy for the Monterey Formation of California. Siliceous Microfossils and Microplankton of the Monterey Formation and Modern Analogs.

[ref-12] Berkoff M, Barnes LG (1988). The evolution of the dusignathines: pseudo-walruses of the late Miocene. PaleoBios.

[ref-13] Berta A, Sumich JL, Kovacs KM (2006). Marine mammals: evolutionary biology.

[ref-14] Boessenecker RW (2013a). Pleistocene survival of an archaic dwarf baleen whale (Mysticeti: Cetotheriidae). Naturwissenschaften.

[ref-15] Boessenecker RW (2013b). A new marine vertebrate assemblage from the Late Neogene Purisima Formation in Central California, part II: pinnipeds and cetaceans. Geodiversitas.

[ref-16] Boessenecker RW (2017). A new early Pliocene record of the toothless walrus *Valenictus* (Carnivora, Odobenidae) from the Purisima Formation of Northern California. PaleoBios.

[ref-17] Boessenecker SJ, Boessenecker RW, Geisler JH (2018). Youngest record of the extinct walrus *Ontocetus emmonsi* from the Early Pleistocene of South Carolina and a review of North Atlantic walrus biochronology. Acta Palaeontologica Polonica.

[ref-18] Boessenecker RW, Churchill M (2013). A reevaluation of the morphology, paleoecology, and phylogenetic relationships of the enigmatic walrus *Pelagiarctos*. PLOS ONE.

[ref-19] Boessenecker RW, Churchill M (2015). The oldest known fur seal. Biology Letters.

[ref-20] Boessenecker RW, Churchill M (2018). The last of the desmatophocid seals: a new species of *Allodesmus* from the upper Miocene of Washington, USA, and a revision of the taxonomy of Desmatophocidae. Zoological Journal of the Linnean Society.

[ref-21] Boessenecker RW, Perry FA, Schmitt JG (2014). Comparative taphonomy, taphofacies, and bonebeds of the Mio-Pliocene Purisima Formations, Central California: strong physical control on marine vertebrate preservation in shallow marine settings. PLOS ONE.

[ref-22] Bowdich TE (1821). An analysis of the natural classification of mammalia.

[ref-23] Cantino PD, De Queiroz K (2014). www.ohiou.edu/phylocode.

[ref-24] Churchill M, Clementz MT, Kohno N (2015). Cope’s rule and the evolution of body size in Pinnipedimorpha (Mammalia: Carnivora). Evolution.

[ref-25] Cohen KM, Harper DAT, Gibbard PL (2017). ICS International Chronostratigraphic Chart 2017/02. International Commission on Stratigraphy, IUGS. www.stratigraphy.org.

[ref-26] Debey LB, Pyenson ND (2013). Osteological correlates and phylogenetic analysis of deep diving in living and extinct pinnipeds: what good are big eyes?. Marine Mammal Science.

[ref-27] De Blainville HMD (1820). Surquelques cranes de phoques. Journal de Physique de Chimie, d’Histroire Naturelle et des Arts.

[ref-28] De Christol J (1832). Description of *Hipparion* (not title). Annales Science l’Industrie du midi de la France.

[ref-29] Deméré TA, Larue DK, Steel RJ (1983). The Neogene San Diego Basin: a review of the marine Pliocene San Diego Formation. Cenozoic Marine Sedimentation Pacific Margin, U.S.A.

[ref-30] Deméré TA (1994a). Two new species of fossil walruses (Pinnipedia: Odobenidae) from the Upper Pliocene San Diego Formation, California. Proceedings of the San Diego Society of Natural History.

[ref-31] Deméré TA (1994b). The Family Odobenidae: a phylogenetic analysis of fossil and living taxa. Proceedings of the San Diego Society of Natural History.

[ref-32] Deméré TA, Berta A (2001). A reevaluation of *Proneotherium repenningi* from the Miocene Astoria Formation of Oregon and its position as a basal odobenid (Pinnipedia: Mammalia). Journal of Vertebrate Paleontology.

[ref-130] Deméré TA, Berta A (2005). New skeletal material of *Thalassoleon* (Otariidae: Pinnipedia) from the late Miocene-Early Pliocene (Hemphillian) of California. Bulletin of the Florida Museum of Natural History.

[ref-33] Deméré TA, Berta A, Adam PJ (2003). Pinnipedimorph evolutionary biogeography. Bulletin of the American Museum of Natural History.

[ref-34] Domning DP (1994). A phylogenetic analysis of the Sirenia. Proceedings of the San Diego Society of Natural History.

[ref-35] Dorsey RJ, Housen BA, Janecke SU, Fanning M, Spears ALF (2011). Stratigraphic record of basin development within the San Andreas fault system: Late Cenozoic Fish Creek-Vallecito Basin, Southern California. Geological Society of America Bulletin.

[ref-36] Dubrovo IA (1981). A new subfamily of fossil seals (Pinnipedia, Kamtschatarctinae subfam. nov.). Proceedings of the Academy of Sciences of the USSR.

[ref-37] Du Bus B (1867). Sur quelques mammiferes du crag d’Anvers. Bulletins de L’Academie Royale des Sciences, des Lettres et des Beaux Arts.

[ref-38] Dyke AS, Hooper J, Harington CR, Savelle JM (1999). The Late Wisconsinan and Holocene Record of Walrus (*Odobenus rosmarus*) from North America: a review with new data from arctic and Atlantic Canada. Arctic.

[ref-39] Fay FH (1982). Ecology and biology of the Pacific walrus, *Odobenus rosmarus divergens* Illiger. North American Fauna.

[ref-40] Gill T (1866). Prodrome of a monograph of the pinnipedes. Proceedings of the Essex Institute.

[ref-41] Gladenkov YB (2013). Cenozoic orogenic phases in the northwestern framing of the Pacific. Stratigraphy and Geological Correlation.

[ref-42] Gradstein FM, Ogg JG, Schmitz M, Ogg G (2012). The Geologic Time Scale 2012.

[ref-43] Gradstein FM, Ogg JG, Smith AG, Bleeker W, Lourens LJ (2004). A new Geologic Time Scale, with special reference to Precambrian and Neogene. Episodes.

[ref-44] Harington CR (1984). Quaternary marine and land mammals and their paleoenvironmental implications–some examples from northern North America. Carnegie Museum of Natural History.

[ref-45] Harington CR, Beard G (1992). The Qualicum Walrus: a late Pleistocene walrus (*Odobenus rosmarus*) skeleton from Vancouver Island, British Columbia, Canada. Annales Zoologici Fennici.

[ref-46] Hilgen FJ, Lourens LJ, Van Dam JA, Gradstein FM, Ogg JG, Schmitz M, Ogg G (2012). The Neogene period. Geologic Time Scale.

[ref-47] Horikawa H (1995). A primitive odobenine walrus of Early Pliocene age from Japan. Island Arc.

[ref-48] Hosford Scheirer AH, Magoon LB (2007). Age, distribution, and stratigraphic relationship of rock units in the San Joaquin Basin province, California. U.S. Geological Survey Professional Paper.

[ref-49] Howell AB (1925). Asymmetry in the skulls of mammals. Proceedings of the United States National Museum.

[ref-50] Ito M, Katsura Y (1992). Inferred glacio-eustatic control for high-frequency depositional sequences of the Plio-Pleistocene Kazusa Group, a forearc basin fill in Boso Peninsula, Japan. Sedimentary Geology.

[ref-51] Kastelein RA, Gerrits NM, Dubbeldam JL (1991). The anatomy of the walrus head (*Odobenus rosmarus*) Part 2: description of the muscles and of their role in feeding and haul-out behaviour. Aquatic Mammals.

[ref-52] Kazaoka O, Suganuma Y, Okada M, Kameo K, Head MJ, Yoshida T, Sugaya M, Kameyama S, Ogitsu I, Nirei H, Aida N, Kumai H (2015). Stratigraphy of the Kasuza Group, Boso Peninsula: an expanded and highly-resolved marine sedimentary record from the lower and middle Pleistocene of Central Japan. Quaternary International.

[ref-53] Kellogg R (1921). A new pinniped from the Upper Pliocene of California. Journal of Mammalogy.

[ref-54] Kellogg R (1922). Pinnipeds from Miocene and Pliocene deposits of California and a résumé of current theories regarding the origin of Pinnipedia. University of California Publications in Geological Sciences.

[ref-55] Kellogg R (1927). Fossil pinnipeds from California. Carnegie Institution of Washington Publication.

[ref-56] Kellogg R (1931). Pelagic mammals from the Temblor Formation of the Kern River region, California. Proceedings of the California Academy of Sciences.

[ref-57] Kennett JP, Rozo-Vera GA, Machain Castillo ML (2000). Latest Neogene planktonic foraminiferal biostratigraphy of the California Margin. Proceedings of the Ocean Drilling Program.

[ref-58] Kloess PA, Parham JF (2017). A specimen-based approach to reconstructing the late Neogene seabird communities of California. Palaeogeography Palaeoclimatology Palaeoecology.

[ref-59] Kohno N (1994). A new Miocene pinniped in the genus *Prototaria* (Carnivora: Odobenidae) from the Moniwa Formation, Miyagi, Japan. Journal of Vertebrate Paleontology.

[ref-60] Kohno N (2006). A new Miocene odobenid (Mammalia: Carnivora) from Hokkaido, Japan, and its implications for odobenid phylogeny. Journal of Vertebrate Paleontology.

[ref-61] Kohno N, Barnes LG, Hirota K (1995). Miocene fossil pinnipeds of the genera *Prototaria* and *Neotherium* (Carnivora; Otariidae; Imagotariinae) in the North Pacific Ocean: evolution, relationships and distribution. Island Arc.

[ref-62] Kohno N, Narita K, Koike H (1998). An early Pliocene odobenid (Mammalia: Carnivora) from the Joshita Formation, Nagano Prefecture, central Japan. Research Reports of the Shinshushinmachi Fossil Museum.

[ref-63] Kohno N, Ray CE (2008). Pliocene walruses from the Yorktown Formation of Virginia and North Carolina, and a systematic revision of the North Atlantic Pliocene walruses. Virginia Museum of Natural History Special Publication.

[ref-64] Kohno N, Tomida Y, Hasegawa Y, Furusawa H (1995). Pliocene tusked odobenids (Mammalia: Carnivora) in the Western North Pacific, and their paleobiogeography. Bulletin of the National Science Museum, Tokyo Series C (Geology & Paleontology).

[ref-65] Kucera M, Kennett JP (2000). Biochronology and evolutionary implications of Late Neogene California planktonic foraminiferal events. Marine Micropaleontology.

[ref-66] Leidy J (1859). Remarks on *Dromatherium sylvestre* and *Ontocetus emmonsi*. Proceedings of the Academy of Natural Sciences.

[ref-67] Linnaeus C (1758). Systema naturae per regna tria naturae, secundum classes, ordines, genera, species, cum characteribus, differentiis, synonymis, locis. Editio Decima.

[ref-108] Lyon GM (1941). A Miocene sea lion from Lomita, California. University of California Publications in Zoology.

[ref-68] Loomis KB, Kuespert JG, Reid SA (1990). Depositional environments and sedimentary history of the Etchegoin Group, West-central San Joaquin Valley, California. Structure, Stratigraphy, and Hydrocarbon Occurrences of the San Joaquin Basin, California.

[ref-69] Loomis KB (1992). New K-Ar Ages from the tuffs in the Etchegoin Formation, San Joaquin Basin, California. Isochron/West.

[ref-70] Lydersen C, Würsig B, Thewissen JGM, Kovacs KM (2018). Walrus. Encyclopedia of Marine Mammals.

[ref-109] Merriam JC (1915). New horses from the Miocene and Pliocene of California. University of California Publications, Bulletin of the Department of Geology.

[ref-71] Miller EH, Würsig B, Thewissen JGM, Kovacs KM (2018). Baculum. Encyclopedia of Marine Mammals.

[ref-72] Mitchell ED (1961). A new walrus from the imperial Pliocene of Southern California: with notes on odobenid and otariid humeri. Contributions in Science, Natural History Museum of Los Angeles County.

[ref-73] Mitchell ED (1966). The Miocene pinniped *Allodesmus*. University of California Publications in Geological Sciences.

[ref-74] Mitchell ED (1968). The Mio-Pliocene pinniped *Imagotaria*. Journal of the Fisheries Research Board of Canada.

[ref-110] Miyazaki S, Horikawa H, Kohno N, Hirota K, Kimura M, Hasegawa Y, Tomida Y, Barnes LG, Ray CE (1995). Summary of the fossil record of pinnipeds of Japan, and comparisons with that from the eastern North Pacific. Island Arc.

[ref-75] Morgan GS (1994). Miocene and Pliocene marine mammal faunas from the Bone Valley Formation of Central Florida. Proceedings of the San Diego Society of Natural History.

[ref-76] Nakagawa M (1994). Late Miocene Imagotarinae from the Saigawa-River Nagano City, central Japan. Monograph of the Association for the Geological Collaboration in Japan.

[ref-77] Parham JF, Pyenson ND (2010). New sea turtle from the Miocene of Peru and the iterative evolution of feeding ecomorphologies since the Cretaceous. Journal of Paleontology.

[ref-78] Péron F (1816). Voyage de découvertes aux terres australes, exécuté sur les Corvettes le Géographe, le Naturaliste, et al Goëlette le Casuarina, pendent les années 1800, 1801, 1802, 1803 et 1804. L’Imprimerie Royale, Paris.

[ref-79] Pimiento C, Griffin JN, Clements CF, Silvestro D, Varela S, Uhen MD, Jaramillo C (2017). The Pliocene marine megafauna extinction and its impact on functional diversity. Nature Ecology & Evolution.

[ref-80] Prothero DR, Bitboul CZ, Moore GW, Moore EJ (2001). Magnetic stratigraphy of the lower and middle Miocene Astoria Formation, Lincoln County, Oregon. Pacific Section SEPM Special Publication.

[ref-81] Prothero DR, Francisco S, Denke LL (2008). Magnetic stratigraphy of the Early to Middle Miocene Olcese Sand and Round Mountain Silt, Kern County, California. New Mexico Museum and Natural History & Science Bulletin.

[ref-82] Prothero DR, Lau JN, Armentrout JM (2001). Magnetic stratigraphy of the upper Miocene (Wishkahan) Empire Formation, Coos County, Oregon. Magnetic Stratigraphy of the Pacific Coast Cenozoic. Pacific Section SEPM.

[ref-83] Pyenson ND, Irmis RB, Lipps JH, Barnes LG, Mitchell ED, McLeod SA (2009). Origin of a widespread marine bonebed deposited during the middle Miocene Climatic Optimum. Geology.

[ref-84] Raschke RE (1984). Early and middle Miocene vertebrates from the Santa Ana Mountains, California. Memoirs of the Natural History Foundation of Orange County.

[ref-85] Repenning CA, Tedford RH (1977). Otarioid seals of the Neogene. U.S. Professional Paper.

[ref-86] Sarna-Wojcicki AM, Deino AL, Fleck RJ, McLaughlin RJ, Wagner D, Wan E, Wahl D, Hillhouse JW, Perkins M (2011). Age, composition, and areal distribution of the Pliocene Lawlor Tuff, and three younger Pliocene tuffs, California and Nevada. Geosphere.

[ref-87] Sawlan MG, Smith JG (1984). Petrologic characteristics, age and tectonic setting of the Neogene volcanic rocks in northern Baja California Sur, Mexico. In Frizzel VA, ed. Geology of the Baja California Peninsula. Pacific Section, Society of Economic Paleontologists and Minerologists Publication.

[ref-88] Sha[h]bazi S, Magallanes I, Parham JF, Boessenecker RW (2016). Fossil Walrus skulls from the Empire Formation of Oregon represent a new lineage from the late Miocene and support a significant late Miocene radiation of Odobenids. Journal of Vertebrate Paleontology, Program and Abstracts.

[ref-89] Shilo NA, Minyuk PS (2006). Magnetochronology of the Climatic Optimum at the Early–Middle Miocene Transition in Northeast Russia. Doklady Earth Sciences.

[ref-90] Sivertsen E (1954). A survey of eared seals (family Otariidae) with remarks on the Antarctic seals collected by N/K “Norvegia” in 1928–1929. Det Norske Videnkaps-Akademii Oslo.

[ref-91] Squires RL (1991). New Morphologic and Stratigraphic Data on *Calyptogena* (*Calyptogena*) *gibbera* Crickmay, 1929 (Bivalvia: Vesicomyidae) from the Pliocene and Pleistocene of Southern California. Veliger.

[ref-92] Swofford DL (2002). PAUP*. Phylogenetic Analysis Using Parsimony (*and Other Methods).

[ref-93] Takano O (1990). Depositional process of trough-fill turbidites of the upper Miocene to Pliocene Tamugigawa Formation, Northern Fossa Magna, central Japan. Journal of the Geological Society of Japan.

[ref-94] Takano O, Hoyanagi K, Noto M, Ota K, Yahata M, Sedimentary Facies Group of the Kabato Collaborative Research Group (1996). Formation processes of depositional sequences of the Neo-gene System in the southern part of the Kabato Mountains, Hokkaido, Japan. Earth Science (Chikyu Kagaku).

[ref-95] Takeyama K, Ozawa T (1984). A new Miocene otarioid from Japan. Proceedings of the Japan Academy, Series B.

[ref-96] Tanaka Y, Kohno N (2015). A new Late Miocene odobenid (Mammalia: Carnivora) from Hokkaido, Japan suggests rapid diversification of basal Miocene odobenids. PLOS ONE.

[ref-111] Tedford RH, Albright LB, Barnosky AD, Ferrusquía-Villafranca I, Hunt RM, Storer JE, Swisher CC, Voorhies MR, Webb SD, Whistler DP, Woodburne MO (2004). Mammalian biochronology of the Arikareean through Hemphillian interval (late Oligocene through early Pliocene Epochs). Late Cretaceous and Cenozoic mammals of North America: Biostratigraphy and geochronology.

[ref-97] Tonomori W, Sawamura H, Sato T, Kohno N (2018). A new Miocene pinniped *Allodesmus* (Mammalia: Carnivora) from Hokkaido, northern Japan. Royal Society Open Science.

[ref-98] True FW (1905). Diagnosis of a new genus and species of fossil sea-lion from the Miocene of Oregon. Smithsonian Miscellaneous Collections.

[ref-99] Tsuji T, Saito Y, Ikuji Y, Ichinoseki T, Obuse A, Kai K, Yanagimoto Y (1992). Sedimentary environments and sequences of the Ponsubetsu and Subetsu Formations of the Minenobu Gas Field, northern Ishikari plain, central Hokkaido. Research Reports of the Japan Petroleum Exploration Research Center.

[ref-100] Velez-Juarbe J (2017). *Eotaria citrica*, sp. nov., a new stem otariid from the “Topanga” Formation of Southern California. PeerJ.

[ref-101] Velez-Juarbe J (in press). New data on the early odobenid *Neotherium mirum* Kellogg, 1931, and other pinniped remains from the Sharktooth Hill Bonebed, California. Journal of Vertebrate Paleontology.

[ref-102] Velez-Juarbe J, Salinas-Márquez FM (2018). A dwarf walrus from the Miocene of Baja California Sur, Mexico. Royal Society Open Science.

[ref-103] Vélez-Juarbe J, Wood AR, Pimiento C (2016). Pygmy sperm whales (Odontoceti, Kogiidae) from the Pliocene of Florida and North Carolina. Journal of Vertebrate Paleontology.

[ref-104] Vendrasco MJ, Eernise DJ, Powell CL, Fernandez CZ (2012). Polyplacophora (Mollusca) from the San Diego Formation: a remarkable assemblage of fossil chitons from the Pliocene of Southern California. Contributions in Science, Natural History Museum of Los Angeles County.

[ref-105] Webb SD, Hulbert RC, Morgan GS, Evans HF (2008). Terrestrial mammals of the Palmetto Fauna (early Pliocene, latest Hemphillian) from the Central Florida Phosphate District. In: Wang X, Barnes LG, eds. Geology and vertebrate paleontology of western and southern North America, contributions in honor of David P. Whistler. Natural History Museum of Los Angeles County Science Series.

[ref-112] Whitmore FC, Barnes LG (2008). The Herpetocetinae, a new subfamily of extinct baleen whales (Mammalia, Cetacea, Cetotheriidae). Virginia Museum of Natural History Special Publication.

[ref-106] Yeats RS, Huftile GJ, Stitt LT (1994). Late Cenozoic Tectonics of the East Ventura Basin, Transverse Ranges, California. AAPG Bulletin.

